# Transcriptional Patterns in Stages of Alzheimer's Disease Are Cell-Type–Specific and Partially Converge with the Effects of Alcohol Use Disorder in Humans

**DOI:** 10.1523/ENEURO.0118-24.2024

**Published:** 2024-10-09

**Authors:** Arpita Joshi, Federico Manuel Giorgi, Pietro Paolo Sanna

**Affiliations:** ^1^The Scripps Research Institute, San Diego, California 92117; ^2^University of Bologna, Bologna 40136, Italy

**Keywords:** alcohol use disorder, Alzheimer's disease, differential gene expression, gene networks, master regulator analysis, snRNA-seq

## Abstract

Advances in single-cell technologies have led to the discovery and characterization of new brain cell types, which in turn lead to a better understanding of the pathogenesis of Alzheimer's disease (AD). Here, we present a detailed analysis of single-nucleus (sn)RNA-seq data for three stages of AD from middle temporal gyrus and compare it with snRNA-seq data from the prefrontal cortices from individuals with alcohol use disorder (AUD). We observed a significant decrease in both inhibitory and excitatory neurons, in general agreement with previous reports. We observed several cell-type–specific gene expressions and pathway dysregulations that delineate AD stages. Endothelial and vascular leptomeningeal cells showed the greatest degree of gene expression changes. Cell-type–specific evidence of neurodegeneration was seen in multiple neuronal cell types particularly in somatostatin and Layer 5 extratelencephalic neurons, among others. Evidence of inflammatory responses was seen in non-neuronal cells, particularly in intermediate and advanced AD. We observed common perturbations in AD and AUD, particularly in pathways, like transcription, translation, apoptosis, autophagy, calcium signaling, neuroinflammation, and phosphorylation, that imply shared transcriptional pathogenic mechanisms and support the role of excessive alcohol intake in AD progression. Major AUD gene markers form and perturb a network of genes significantly associated with intermediate and advanced AD. Master regulator analysis from AUD gene markers revealed significant correlation with advanced AD of transcription factors that have implications in intellectual disability, neuroinflammation, and other neurodegenerative conditions, further suggesting a shared nexus of transcriptional changes between AD and AUD.

## Significance Statement

This study holds significant implications for understanding the intricate molecular landscape of AD and its intersection with alcohol use disorder (AUD). By profiling transcriptional changes in the neocortex associated with AD progression and comparing them with those in AUD, we shed light on shared gene expression and pathway dysregulations between the two conditions. Our findings corroborate prior research on neuronal depletion and highlight novel insights into cell-type–specific gene expression patterns in AD stages. Moreover, the identification of common genetic signatures suggests a potential exacerbating effect of AUD on AD progression. This comprehensive analysis not only deepens our understanding of AD pathology but also underscores the importance of considering AUD as a potential risk factor for accelerating AD onset or severity.

## Introduction

The Centers for Disease Control and Prevention defines Alzheimer's disease (AD) as the most prevalent form of dementia: one that begins with mild memory loss and progresses into severe brain damage resulting in inability of the individual to carry a conversation and conduct daily routine (CDC, 2022). By the year 2060, ∼14 million people in the United States are projected to be living with AD (Matthews et al., 2019). While aging and genetic predisposition are considered to be the major risk factors, a number of lifestyle factors have also been shown to have a significant effect in the disease onset (Lee et al., 2022; Omura et al., 2022). However, alcohol consumption and AD (and other dementias) have a nuanced relationship, as is reported in *The Lancet* Commission on dementia prevention, intervention, and care (Livingston et al., 2020), wherein both long-term dependence and abstinence can be risk factors, but long-term heavy drinking is a putative catalyst for early-onset AD and its progression (Livingston et al., 2020). In general, there have been studies that associate long-term heavy alcohol intoxication with the onset of AD symptoms and steeper cognitive decline (Harwood et al., 2010; Heymann et al., 2016; Chosy et al., 2022). A large retrospective study found a strong association between alcohol use disorder (AUD) and early-onset dementia (Schwarzinger et al., 2018). In addition, transcriptional evidence in AD mouse models supports that a history of alcohol dependence promotes the earlier onset of cognitive impairment in mice genetically predisposed to AD (Sanna et al., 2023). At the cellular and molecular levels, alcohol affects multiple substrates relevant to AD and other neurodegenerative diseases such as promoting neuroinflammation, oxidative stress, and mitochondrial dysfunction, interfering with autophagy and proteostasis, transcriptional and epigenetic changes, and changes in cerebral blood flow and vessel integrity (Araujo et al., 2021; León et al., 2022; Pierson et al., 2024). However mechanistic insights on the role of excessive alcohol intake on the progression of AD remain elusive.

To our knowledge, the present study is the first investigation that analyzes transcriptomic alterations in both AD and AUD using single-cell resolution in human datasets. We characterized the gene expression profiles of individuals at three clinically defined AD stages from the Seattle Alzheimer's Disease Cell Atlas (SEA-AD) consortium for individuals with AD (Gabitto et al., 2023) which spans the entire gamut of the severity of the disease. We used 85 samples, including control samples and three clinically defined stages of AD. We used a comprehensive definition of an AD stage that includes the neuropathological burden of the disease and all the orthogonal pathologies to ultimately use a clinically accepted staging of AD (see below, Sample stratification for AD stages). We also used advanced deep generative computational tools to extract rich latent representations of the transcriptome to identify differentially expressed genes (DEGs). We found several common findings with (Mathys et al., 2023) study like somatostatin (Sst) neuronal decline and glial apoptosis with AD progression. Likewise, Sun et al. (2023a) elucidated marked dysregulation in cerebrovascular cell types in AD, and in Sun et al. (2023b), authors identified microglial states associated with AD progression, which also parallels our findings of dysregulation in pathways indicative of inflammation in vascular and microglial cell types.

We observed several unique genetic markers and cell-type–specific expression patterns associated with different stages of AD. Our findings highlight disrupted autophagy-related genes in early AD, and later stages show heightened expression of neuroinflammation markers and stress-related neuropeptides in excitatory neurons, along with dysregulated synaptic signaling, transcriptional evidence of neurodegeneration, and cell-death–related pathways in neuronal and glial cells, in general agreement with recent studies (Mathys et al., 2023; Sun et al., 2023a). Additionally, our analysis reveals shared perturbations in genes and pathways between AD and AUD across AD stages. Some noteworthy processes being blood–brain barrier disintegration in vascular cell types via negative regulation of angiogenesis, vasculature development and ECM (extracellular matrix) signaling, and cell-death–related pathways in neuronal cells, including significant overlap in the processes associated with GABAergic cell types.

## Materials and Methods

### SEA-AD

**Figure 1.**
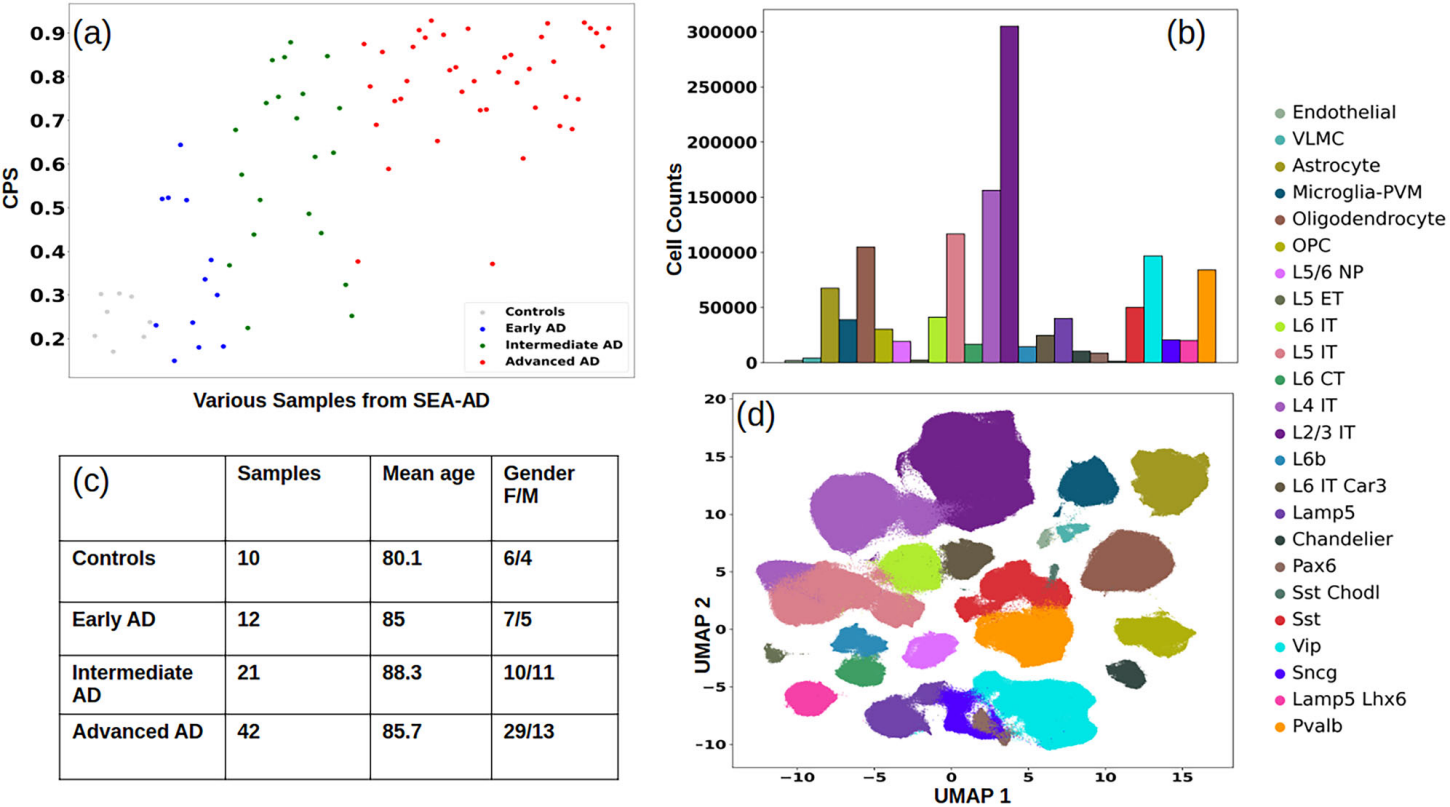
Summary of samples and cells in the publicly available SEA-AD dataset (Gabitto et al., 2023): (***a***) CPS measuring AD progression of the samples used in the work of Gabitto et al. (2023), (***b***) distribution of cell counts of various cell types in the data, (***c***) summary of the samples in each of the AD group and control samples, (***d***) UMAP embedding of samples used in this work.

The SEA-AD consortium (Gabitto et al., 2023) works toward establishing the best practices to analyze and create the richest data repository for AD patients that covers the spectrum of AD pathology and is publicly available. Its recent release offers a high-level cell–type resolution from all stages of the disease from the middle temporal gyrus, an area of the brain involved in language and semantic memory processing (Papeo et al., 2019). It presents AD on a continuous trajectory using multiple data modalities: single-nucleus (sn)RNA-seq, ATAC-seq (assay for transposase accessible chromatin with sequencing), and MERFISH (multiplexed error-robust fluroesence in situ hybridization - spatial transcriptomics imaging data; Cisar et al., 2023). We used the RNA-seq profiles and the meta data of the 85 patients from this study and used their pathological scores to identify the samples belonging to various AD stages. Figure 1*a* shows the progression score of each sample, which does seem to follow the disease progression. Figure 1*b* shows the distribution of various cell types. Figure 1*c* shows a summary of the control samples and the three AD stages. Maximum samples in the cohort are advanced AD cases. The mean age of each group is ∼80 years. There are significantly more females than males in the advanced AD group, which is consistent with the findings of Liampas et al. (2023) which purports greater chances of AD in the female population. Figure 1*d* shows the Uniform Manifold Approximation and Projection (UMAP) embedding of all the samples. All the cell types are from three broad families: (1) non-neuronal, oligodendrocyte, OPC (oligodendrocyte precursor cells), microglia–PVM (perivascular macrophages), astrocyte, endothelial, and VLMC (vascular leptomeningeal cells); (2) neuronal glutamatergic (excitatory) neurons, L2/3 IT (Layer 2/3 intratelencephalic) L4 IT, L5 IT, L6 IT, L6 IT Car3, L6b, L5 ET (extratelencephalic projecting also known as L5 pyramidal tract), L5/6 NP (near-projecting), and L6 CT (corticothalamic); (3) neuronal GABAergic (inhibitory) neurons, Lamp5 (lysosomal-associated membrane protein), Chandelier, Pax6 (paired box-6), Sst, Sst Chodl, Vip (vasoactive intestinal polypeptide), Sncg (synuclein gamma), Lamp5Lhx6, and Pvalb (parvalbumin). We found a decrease in neuronal cell composition through the AD stages, along with several cell-type–specific gene expression changes, that are highlighted in the results section. We also found several biological processes using GSEA (gene set enrichment analysis) that are selectively dysregulated in a cell-type–specific manner.

### Sample stratification for AD stages

The progression of AD is well studied and involves a plethora of composite pathologies (Crystal et al., 1988; Beach et al., 1997; Price and Morris, 1999; Albert et al., 2011; Sperling et al., 2011). Among the first studied characterizing traits that utilize the progression of neurofibrillary tangles (NFTs) in the brain is the Braak staging (Braak and Braak, 1991). It delineates six stages that are often collapsed into four (Braak and Braak, 1991; Nagy et al., 1998): no NFTs, Braak Stages 1/2 with NFTs mostly in the entorhinal cortex, Braak 3/4 with NFTs more abundant in hippocampus and amygdala, and Braak 5/6 with NFTs widely distributed throughout the neocortex. Another major pathologic change is the accumulation of plaques of β-amyloid (Aβ) peptides that define the five Thal phases (Thal et al., 2002; Wharton et al., 2019). The first Thal phase has these deposits in the neocortex, and the second one progresses to the hippocampus and cingulate gyrus, the third phase into the striatum and basal forebrain, the fourth one in the midbrain central gray matter/brainstem, and finally the fifth one into the cerebellum. Another type of Aβ plaque, neuritic plaque, is characterized by dystrophic neurites along with glial activation (Masliah et al., 1993). Consortium to Establish a Registry for AD (CERAD; Mirra et al., 1991) identifies four stages according to the density of these plaques in the neocortex. We use the National Institutes of Aging (NIA)-defined scoring mechanism that combines these scores to define an AD stage, which is discussed next.

Along the three parameters of “ABC” (amyloid, Braak, CERAD) scoring, the NIA has developed quantitative measures for the AD neuropathological change (ADNC; Hyman et al., 2012; Bianchetti et al., 2023). With its inherent scales of “ABC” scores, it identifies four stages: (1) not AD, (2) low, (3) intermediate, and (4) high. In this work, we used all these pathological scores to identify control samples and AD samples from three progressive stages, for all the downstream analyses. For the purpose of the present study, we classified AD cases as early, intermediate, and advanced based on the congruence between the ADNC score's threshold for the “ABC” scoring. Figure 1 shows a summary of the samples from each group we studied in this work.

### Brenner AUD data

**Figure 2.**
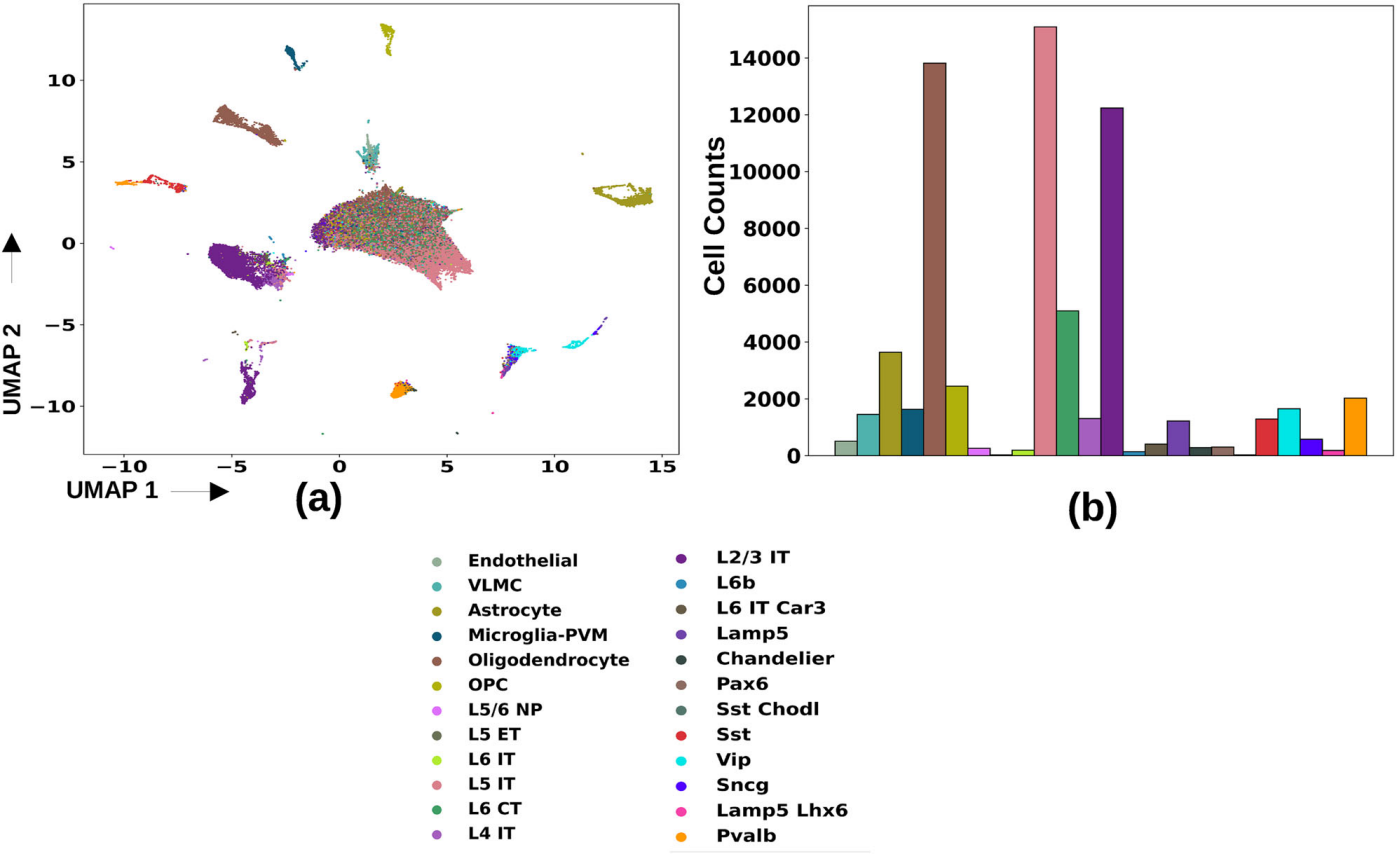
Details of the Brenner AUD dataset (Brenner et al., 2020); we used the Allen Institute's web tool MapMyCells (Allen Institute Tool, 2023) to obtain the same cell-type annotations for this dataset as the SEA-AD data: (***a***) UMAP embedding, (***b***) distribution of cell counts of various cell types in the Brenner AUD data. Extended Data Figure 2-1 shows the cell-type–specific distribution of the log-fold change in maximally dysregulated genes in AUD, and Extended Data Figure 2-2 shows the distribution of number of neurons in the same cell types as in Figure 3.

We used the snRNA-seq data (Brenner et al., 2020) that present gene expression data for four control and three alcohol-dependent individuals. We converted the data to AnnData (Wolf et al., 2018) format and used the Allen Institute's MapMyCells (Allen Institute Tool, 2023) to obtain a cell-type label for each cell in this data. The taxonomy so obtained corresponds with the cell taxonomy of SEA-AD data, shown in Figure 1. The UMAP embedding and the distribution of cell counts of various cell types for this data can be found in Figure 2. In Extended Data Figure 2-1, we show the heatmap of DEGs in the AUD data, and in Extended Data Figure 2-2, we show the distribution of neurons in the corresponding cell types in the two donor categories (controls and AUD).

10.1523/ENEURO.0118-24.2024.f2-1 Figure 2-1 Most significant differentially expressed genes in the various cell types in the AUD data (Brenner et al., 2020). Download Figure 2-1, TIF file.

10.1523/ENEURO.0118-24.2024.f2-2 Figure 2-2 Distribution of number of neurons in the corresponding cell-types in each donor category in AUD data: (a) All Inhibitory Neurons, there is no significant difference between groups (t-test, t(6) = 0.9154, p = 0.3953). (b) All Excitatory Neurons, there is no significant difference between groups (t-test, t(6) = 0.7860, p = 0.4618). (c) Sst, there is no significant difference between groups (t-test, t(6) = 1.333, p = 0.2309). (d) Sst Chodl, there is no significant difference between groups (t-test, t(6) = 0.8563, p = 0.4247). (e) Vip, there is no significant difference between groups (t-test, t(6) = 1.111, p = 0.3093). (f) Sncg, there is no significant difference between groups (t-test, t(6) = 0.6640, p = 0.5314). (g) Pvalb, there is no significant difference between groups (t-test, t(6) = 0.7872, p = 0.4611). (h) L5/6 NP, there is no significant difference between groups (t-test, t(6) = 0.01910, p = 0.9854). (i) L5 ET, there is no significant difference between groups (t-test, t(6) = 0.09232, p = 0.9295). (j) L5 IT, there is no significant difference between groups (t-test, t(6) = 1.152, p = 0.2931). (k) L2/3 IT, there is no significant difference between groups (t-test, t(6) = 0.5118, p = 0.6271). (l) L6 IT Car3, there is no significant difference between groups (t-test, t(6) = 0.3155, p = 0.7631). Download Figure 2-2, TIF file.

### Variational inference for differential gene expression

To analyze the snRNA-seq data used in this work, we used the powerful scVI (single-cell variational inference; Lopez et al., 2018; Virshup et al., 2023) tool, version 1.0.4. It is built on PyTorch (Antiga et al., 2020; Virshup et al., 2023) and AnnData (Wolf et al., 2018) frameworks that use hierarchical Bayesian modeling (Gelman and Hill, 2007) to transform the cell-by-gene data into a latent space and facilitate a number of downstream analysis tasks like clustering, visualization, differential expression, etc. The model itself is essentially a variational autoencoder (Kingma and Welling, 2013) that models the expression values for each cell across genes using a zero-inflated negative binomial distribution. We use an augmentation of this method, lvm-DE (latent variable model, differential expression; Boyeau et al., 2023), which uses a Bayesian formulation of DE hypothesis testing based on log-fold change to avoid lowly expressed genes as differentially expressed. DEGs, shown in Results, were filtered based on whether they were protein coding or not using GENCODE (Frankish et al., 2020) reference annotation, version 44.

### Other bioinformatics tools

We used Python version 3.11.4 and R version 4.3.0. The datasets we used in this work (Brenner et al., 2020; Gabitto et al., 2023) were already studied and preprocessed for quality control. To find the significance of DEGs, we used Python's implementation of GSEA (Subramanian et al., 2005), *gseapy*, version 1.0.6. We used the following collections for pathway analysis: Gene Ontology [Biological Process 2023 (GO: BP)], Cellular Component 2023 (GO: CC), Molecular Function 2023 (GO: MF), “WikiPathway_2023_Human,” “MSigDB_Hallmark_2020,” and “KEGG_2021_Human.” The pathways delineated in GSEA results are filtered at nominal *p* value of 0.01. For master regulator analysis (MRA; Mercatelli et al., 2020), we used “*corto*”, version 1.2.4. We found significantly differentially expressed (FDR corrected *p* ≤ 0.05) genes in AUD and then selected transcription factors among them. Consequently, we observed the network perturbations of these transcription factors in the transcriptional signature of each AD stage.

## Results

Figure 1 presents a summary of the samples studied in this work, and Figure 1*a* shows the trend of continuous pseudoprogression score (CPS) for the samples used in this work. The CPS has been shown by Gabitto et al. (2023) to be congruent with AD progression and ADNC scoring mechanisms. In the following section, we present the cell-type composition of inhibitory and excitatory neurons through the AD stages.

### Neuronal cell decline through AD stages

**Figure 3.**
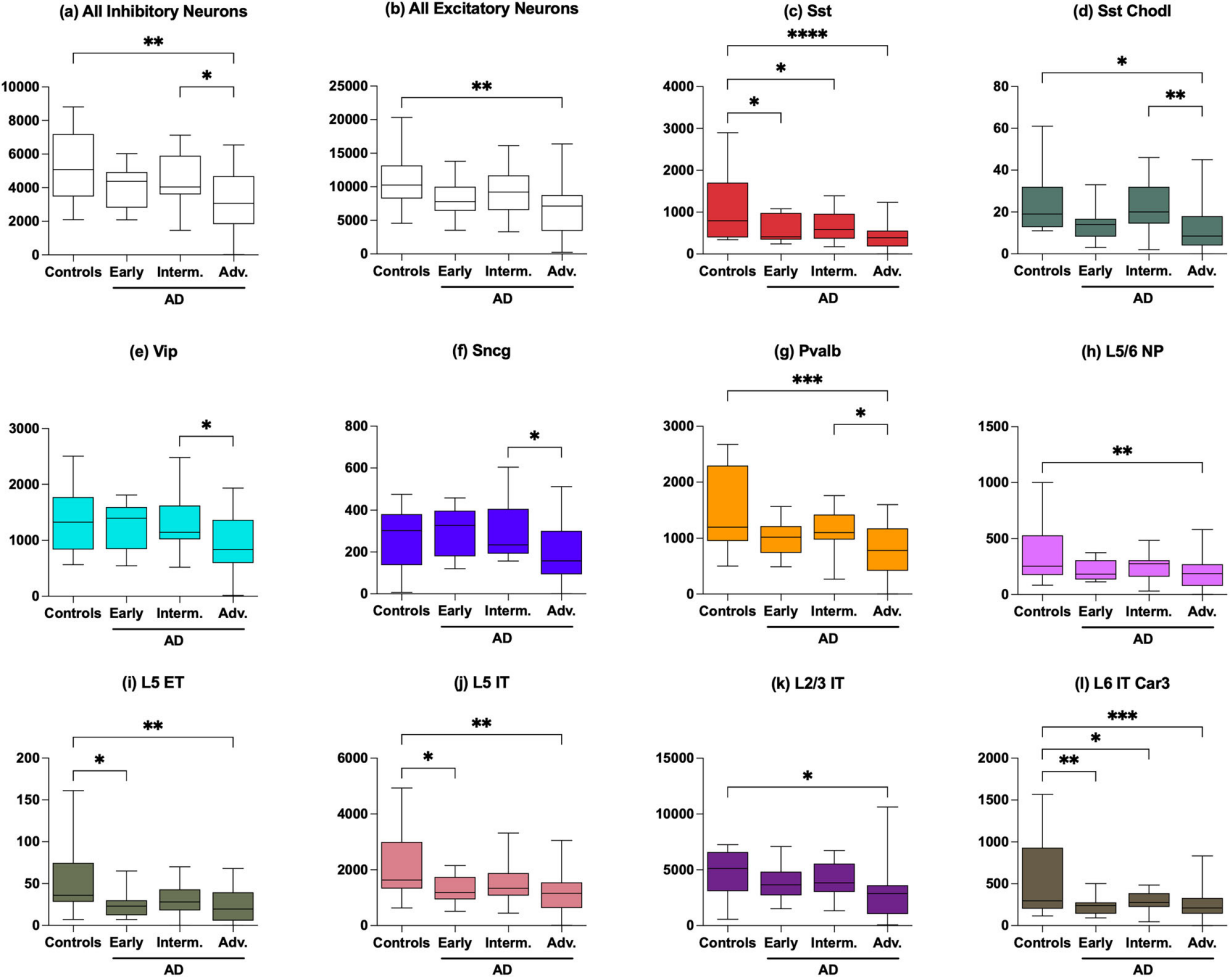
Distribution of number of neurons in the corresponding cell types in each donor category in AD. ***a***, All inhibitory neurons, one-way ANOVA revealed a significant difference between groups (*F*_(3,81)_ = 5.838; *p* = 0.0012). **p < 0.01 versus controls; *p < 0.05 versus intermediate AD; Tukey's test. ***b***, All excitatory neurons, one-way ANOVA revealed a significant difference between groups (*F*_(3,81)_ = 4.364; *p* = 0.0067). **p < 0.01 versus controls; Tukey's test. ***c***, Sst, one-way ANOVA revealed a significant difference between groups (*F*_(3,81)_ = 7.958; *p* = 0.0001). *p < 0.05; ****p < 0.0001 versus controls; Tukey's test. ***d***, Sst Chodl, one-way ANOVA revealed a significant difference between groups (*F*_(3,81)_ = 5.799; *p* = 0.0012). *p < 0.05 versus controls; **p < 0.01 versus intermediate AD; Tukey's test. ***e***, Vip, one-way ANOVA revealed a significant difference between groups (*F*_(3,81)_ = 4.618; *p* = 0.0049). *p < 0.05 versus intermediate AD; Tukey's test. ***f***, Sncg, one-way ANOVA revealed a significant difference between groups (*F*_(3,81)_ = 3.797; *p* = 0.0133). *p < 0.05 versus intermediate AD; Tukey's test. ***g***, Pvalb, one-way ANOVA revealed a significant difference between groups (*F*_(3,81)_ = 7.017; *p* = 0.0003). ***p < 0.001 versus controls; *p < 0.05 versus intermediate AD; Tukey's test. ***h***, L5/6 NP, one-way ANOVA revealed a significant difference between groups (*F*_(3,81)_ = 4.246; *p* = 0.0077). **p < 0.01 versus controls; Tukey's test. ***i***, L5 ET, one-way ANOVA revealed a significant difference between groups (*F*_(3,81)_ = 4.432; *p* = 0.0062). *p < 0.05; **p < 0.01 versus controls; Tukey's test. ***j***, L5 IT, one-way ANOVA revealed a significant difference between groups (*F*_(3,81)_ = 5.403; *p* = 0.0019). *p < 0.05; **p < 0.01 versus controls; Tukey's test. ***k***, L2/3 IT, one-way ANOVA revealed a significant difference between groups (*F*_(3,81)_ = 3.784; *p* = 0.0135). *p < 0.05 versus controls; Tukey's test. ***l***, L6 IT Car3, one-way ANOVA revealed a significant difference between groups (*F*_(3,81)_ = 5.475; *p* = 0.0018). *p < 0.05; **p < 0.01; ***p < 0.001 versus controls; Tukey's test. The distribution of neuronal proportions of these corresponding neuronal categories can be found in Extended Data Figure 3-1.

We observed a decline in the relative abundance of GABAergic/inhibitory and glutamatergic/excitatory neurons compared with control samples through the AD stages (Fig. 3). Within GABAergic neurons, we found a decline in Sst, Sst Chodl, and Pvalb neurons. While we did not segregate samples based on cognitive decline, we observed a general trend of decreasing neurons with the advancing AD stages. Excitatory neurons also significantly declined in advanced AD. Figure 3 summarizes these results for inhibitory and excitatory neurons and their subtypes that have a significant decline. To evaluate the statistical significance for these trends, we performed a one-way ANOVA (analysis of variance) for all the neuronal trends. The neuronal cell types that have significant *p* values (<0.05) are the ones depicted in Figure 3. To further compute the significance of category-wise changes that contribute to the significance of ANOVA *p* values, we performed a post hoc analysis using Tukey's honestly significant differences. For all inhibitory neurons (analyzed together), the trend was significant for changes in control samples versus advanced AD and also among the intermediate versus advanced AD samples. Among excitatory neurons, significant changes were observed only in comparison with controls versus advanced AD samples. Among Sst inhibitory neurons, the changes in post hoc *p* values were significant between control samples and all three AD stages, consistent with Mathys et al. (2023). Other affected inhibitory neurons were Sst Chodl, Pvalb, Vip, and Sncg (Fig. 3*c*–*g*). Deep layers’ excitatory neurons such as L5 IT, L5 ET, and L6 IT Car3 were affected in the progression of the disease, while the superficial layers’ excitatory neurons like L2/3 IT were affected in advanced AD. Diminishing of both Sst and IT neurons with cognitive decline has been reported by Consens et al. (2022). Additionally, in Extended Data Figure 3-1, we show the proportion of these cell types, which shows the distribution of the ratio of the corresponding cell type to that of the total number of cells collected from the individual.

10.1523/ENEURO.0118-24.2024.f3-1 Figure 3-1 Distribution of neuronal proportions in the corresponding neuron types in each donor category in SEA-AD data: (a) All Inhibitory Neurons, there is no significant difference between groups (One-way ANOVA, F(3,81) = 1.053, p = 0.3738). (b) All Excitatory Neurons, there is no significant difference between groups (One-way ANOVA, F(3,81) = 1.339, p = 0.2675). (c) Sst, One-way ANOVA revealed a significant difference between groups (F(3,81) = 4.381, p = 0.0066). **p < 0.01, vs Controls; Tukey's test. (d) Sst Chodl, One-way ANOVA revealed a significant difference between groups (F(3,81) = 4.956, p = 0.0033). **p < 0.01, vs Controls; Tukey's test. (e) Vip, there is no significant difference between groups (One-way ANOVA, F(3,81) = 0.9486, p = 0.4212). (f) Sncg, there is no significant difference between groups (One-way ANOVA, F(3,81) = 1.523, p = 0.2148). (g) Pvalb, One-way ANOVA revealed a significant difference between groups (F(3,81) = 3.697, p = 0.0151). *p < 0.05, vs Controls; Tukey's test. (h) L5/6 NP, there is no significant difference between groups (One-way ANOVA, F(3,81)= 1.168, p = 0.3271). (i) L5 ET, there is no significant difference between groups (One-way ANOVA, F(3,81) = 1.936, p = 0.1303). (j) L5 IT, there is no significant difference between groups (One-way ANOVA, F(3,81) = 2.266, p = 0.0870). (k) L2/3 IT, there is no significant difference between groups (One-way ANOVA, F(3,81) = 2.543, p = 0.0620). (l) L6 IT Car3, there is no significant difference between groups (One-way ANOVA, F(3,81) = 2.377, p = 0.0760). Download Figure 3-1, TIF file.

### DEGs in AD stages

**Figure 4.**
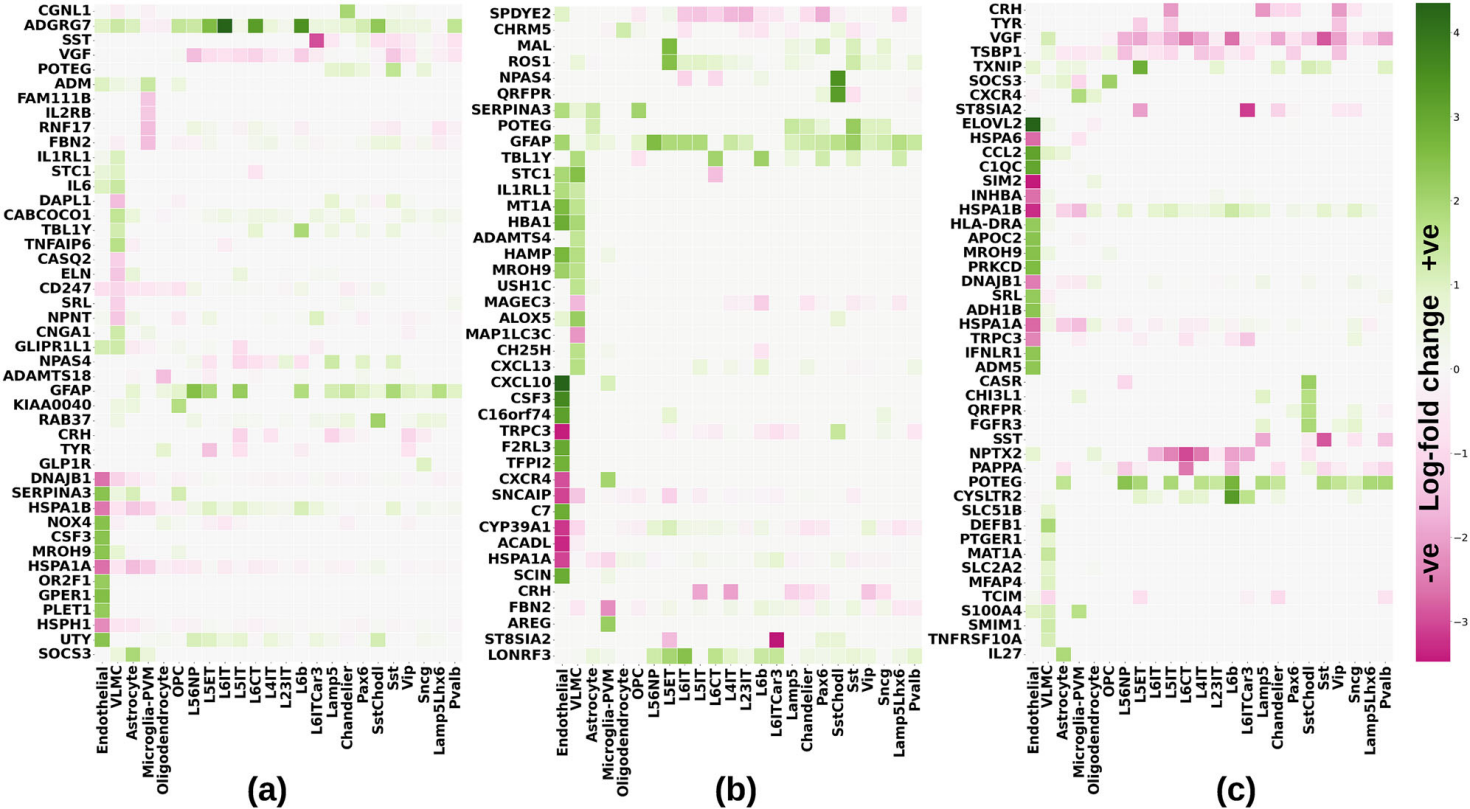
Heatmaps showing the cell-type–specific distribution of the maximally dysregulated genes in (***a***) early AD, (***b***) intermediate AD, (***c***) advanced AD. The volcano plots depicting a subset of these genes can be found in Extended Data Figure 4-1. The whole list of DEGs in the three stages can be found in the Extended Data Tables 4-1–4-3.

We trained the scVI model (Lopez et al., 2018) with preoptimized default hyperparameters for single-cell data on SEA-AD. For each AD stage, we show the most significantly dysregulated genes in Figure 4 via the heatmaps of log-fold change; the corresponding volcano plots for the three AD stages can be found in Extended Data Figure 4-1. The most dysregulated cell types are endothelial and VLMC in all AD stages, at least among the most significant marker genes, evident from Figure 4. The following are the key observations for each AD stage.

10.1523/ENEURO.0118-24.2024.f4-1 Figure 4-1 Volcano plots of some selected DEGs in the three AD stages: (a) Early AD, (b) Intermediate AD, (c) Advanced AD. Download Figure 4-1, TIF file.

10.1523/ENEURO.0118-24.2024.t4-1 Table 4-1 List of DEGs in early AD. Download Table 4-1, XLS file.

10.1523/ENEURO.0118-24.2024.t4-2 Table 4-2 List of DEGs in intermediate AD. Download Table 4-2, XLS file.

10.1523/ENEURO.0118-24.2024.t4-3 Table 4-3 List of DEGs in advanced AD. Download Table 4-3, XLS file.

#### Early AD

Figure 4*a* shows the heatmap of log-fold change for the most significant early AD gene markers. The complete list of DEGs for each cell type obtained as a result of scVI model's *differential_expression* module can be found in Extended Data Table 4-1. Among the top significantly differentially expressed markers are *ADGRG7* (adhesion G-protein–coupled receptor G7), *ADAMTS18*, *CABCOCO1*, *DAPL1*, *DNAJB1*, *ELN*, *HSPA1A*, *IL6*, *KIAA004*, and *TNFAIP6*. Inflammation-related genes are among the significant markers like the proinflammatory cytokine *IL6* (interleukin 6) that is associated with brain atrophy in AD (Capogna et al., 2023), *IL2RB* that is a part of the IL-2 receptor complex, *CD247* that is known to cause immune reactions in the peripheral blood lymphocytes in AD patients (Ikeda et al., 1991; Xu and Jia, 2021), and *IL1RL1* that encodes for soluble ST2 (sST2), a decoy receptor of interleukin-33–ST2 signaling, which has been identified as a genetic risk factor for AD (Jiang et al., 2022). Another major marker unique to early AD is *ADGRG7*, which is significantly upregulated in multiple cell types, maximum in L6 IT neurons (log-fold change, 4.2), and among inhibitory neurons, Sst Chodl. It is upregulated in most glutamatergic cells and is involved in adenylate cyclase-activating G-protein–coupled receptor (GPCR) signaling pathway in early AD, as can be seen in Extended Data Figure 6-1, and GPCR kinases have been implicated to be associated with AD (Guimarães et al., 2021). We found *ADAMTS18* (a disintegrin and metalloproteinase with thrombospondin motifs) to be significantly downregulated in oligodendrocytes, while Ramamurthy et al. (2023) found significant increase in its transcription in the dorsolateral prefrontal cortex in AD; *CABCOO1* (ciliary-associated calcium-binding coiled–coil protein) is significantly upregulated in VLMCs, supported by Extended Data Figure 6-1, which indicates dysregulation of calcium signaling pathway. We found Rab genes (known to regulate autophagy by the Rab GTPase network; Ao et al., 2014), *RAB37* and *RAB38*, are significantly upregulated in early AD (log-fold change of 2.02 in Sst Chodl cells and 2.03 in endothelial cells, respectively); autophagy-related genes were also observed in Mathys et al. (2023) to be associated more with early AD changes relative to late AD. These genes are not dysregulated in advanced AD, while intermediate AD has only *RAB37* upregulated with 0.45 log-fold change.

#### Intermediate AD

This stage is characterized by increased dysregulation (in terms of log-fold changes compared with early AD) among the DEGs in both endothelial cells and VLMCs. Figure 4*b* shows the major marker genes for this AD stage, which includes *NPAS4* (neuronal PAS domain protein 4), *SERPINA3*, *TBL1Y*, *STC1* (stanniocalcin 1), *HBA1*, *ALOX5*, *CXCL10*, *CSF3*, *TRPC3* (transient receptor potential canonical 3), *FBN2*, *AREG*, *ST8SIA2*, *LONRF3*, and *CHRM5*. For the complete list of DEGs in intermediate AD, see Extended Data Table 4-2. Increased chemokine *CXCL10* in the cerebrospinal fluid has been known to be positively correlated with cognitive decline in AD (Galimberti et al., 2006). *CXCR4*, *a* chemokine receptor, also of the C-X-C family, was found to be significantly downregulated (log-fold change, −2.49). We found *NPAS4* to be a significant cell type specifically dysregulated in intermediate AD, with upregulation (log-fold change, 2.88) in GABAergic Sst Chodl and downregulation (log-fold change, −0.83) in glutamatergic L6 CT cells; it was not differentially expressed in advanced AD. *NPAS4* is known to dysregulate excitatory/inhibitory neuronal imbalance, critical for homeostatic synaptic signaling in neurodegenerative diseases, including AD (Fu et al., 2020; Unger et al., 2020), and Opsomer et al. (2020) have found it to decrease along the advancing AD pathology. *TRPC3* is another significantly downregulated gene in intermediate AD, which is known to be enriched in the central nervous system (Li et al., 1999). Similarly, Yamamoto et al. (2007) report that dysregulation of *RPC3* via *BDNF* (brain-derived neurotrophic receptor) plays a role in deregulating tau protein in AD; BDNF is itself only downregulated majorly in intermediate AD (log-fold change, −0.89 and −0.84 in endothelial and glutamatergic L5 IT cells, respectively, also in early AD with a log-fold change of −0.78, but only in L5 IT cells) and not differentially expressed in advanced AD. *ST8SIA2* is known to regulate neuronal migration, axon guidance, and synaptic plasticity (Kamien et al., 2014) and has in general been reported to contribute to cognitive decline (Bao et al., 2022).

#### Advanced AD

Dysregulation of differential expression markers of endothelial cell types is further exacerbated in advanced AD, while the dysregulation in VLMC types appears to be reduced. Furthermore, markers in GABAergic and glutamatergic cell types are considerably more downregulated, like corticotropin-releasing hormone (*CRH*), nerve growth factor (*VGF*), *TYR*, and *ST8SIA2*. *SOCS3* (suppressor of cytokine signaling 3), *ELOVL2* (elongation of very-long-chain fatty acids), *CCL2*, *SIM2*, *CASR*, *DEFB1*, *MAT1A*, and *NPTX2* are among the other major markers. Figure 4*c* shows the most significant DEGs in advanced AD. All the DEGs across cell types can be found in Extended Data Table 4-3. We found *SOCS3* upregulated in astrocytes in all the AD stages, with the maximum in the OPCs in advanced AD, with a log-fold change of 2.1. Levels of expression of *SOCS3* have been found to be elevated in AD brains by multiple studies (Cao et al., 2018; Lyra e Silva et al., 2021), via participation in proinflammatory IL-6 pathway (Lyra e Silva et al., 2021); it also modulates insulin signaling and has been found to be positively correlated with Aβ deposits in the brain (Iwahara et al., 2017; Cao et al., 2018). In addition, we also found it to be upregulated in VLMCs consistent with Sun et al. (2023a) who found it upregulated in the capillary venule endothelial cells of AD patients. Dysregulation of *ELOVL2* has been reported to be a risk factor for AD (Hammouda et al., 2020). Joly-Amado et al. (2020) demonstrated that cerebral overexpression of *CCL2* promotes accumulation of tau protein accumulation *in mouse models*. We found a number of heat shock proteins (HSPs), namely, *HSPA6*, *HSPA1A*, and *HSPA1B*, molecular chaperones, to be significantly downregulated in non-neuronal cells of all AD stages, but the maximum log-fold change was found in advanced AD (in intermediate AD, *HSPA6* was also found to be significantly upregulated in L6 IT Car3 cells, with a log-fold change of 1.23). HSPs have been shown to be of particular relevance in AD (Marino Gammazza et al., 2016) and are in fact considered biomarkers of AD prognosis (Dong et al., 2022). Interestingly, we found *SIM2* (single-minded) significantly downregulated in all AD stages, but the maximum log-fold change of −3.57 was found in the endothelial cells of advanced AD (Fig. 4*c*). *SIM2* is a transcription factor involved in neurogenesis that is implicated in the pathogenesis of Down's syndrome (Muenke et al., 1995; Dahmane et al., 1998). Among the genetic risk factors for AD, the apolipoprotein E gene, *APOE*, and its ensuing cluster of genes are well established (Yu et al., 2007; Bekris et al., 2008; Cervantes et al., 2011; Cruchaga et al., 2012). We found *APOC2*, which is located on chromosome 19 in a cluster with *APOE*, to be significantly upregulated in advanced AD (Fig. 4*c*). DNA methylation of regulatory element in the TOMM40-APOE-APOC2 gene region correlates with AD disease progression (Shao et al., 2018). In addition, we found *REM1* (triggering receptors expressed on myeloid cells) to be significantly downregulated exclusively in advanced AD (Extended Data Table 4-3). *TREM1* is in the same gene cluster as *TREM2* and binds lipidated APOE (Colonna, 2023). Another interesting finding in this stage was *NPTX2* (neuronal pentraxin) that is a known synaptic protein and has been recognized as a prognostic biomarker of AD (Xiao et al., 2017; Libiger et al., 2020). We found *NPTX2* to be upregulated majorly in early AD in endothelial cells; this upregulation in endothelial cells decreases with intermediate and advanced AD, but it is significantly downregulated in multiple glutamatergic cells in advanced AD (maximum in L6 CT with log-fold change of −3.1); in apparent agreement, Soldan et al. (2023) showed that *NPTX2* in the cerebrospinal fluid of AD patients is an early prognostic biomarker of mild cognitive impairment; Mathys et al. (2023) also reported *NPTX2* to be significantly downregulated in individuals with advanced cognitive decline.

#### Progression of marker genes through the AD stages

**Figure 5.**
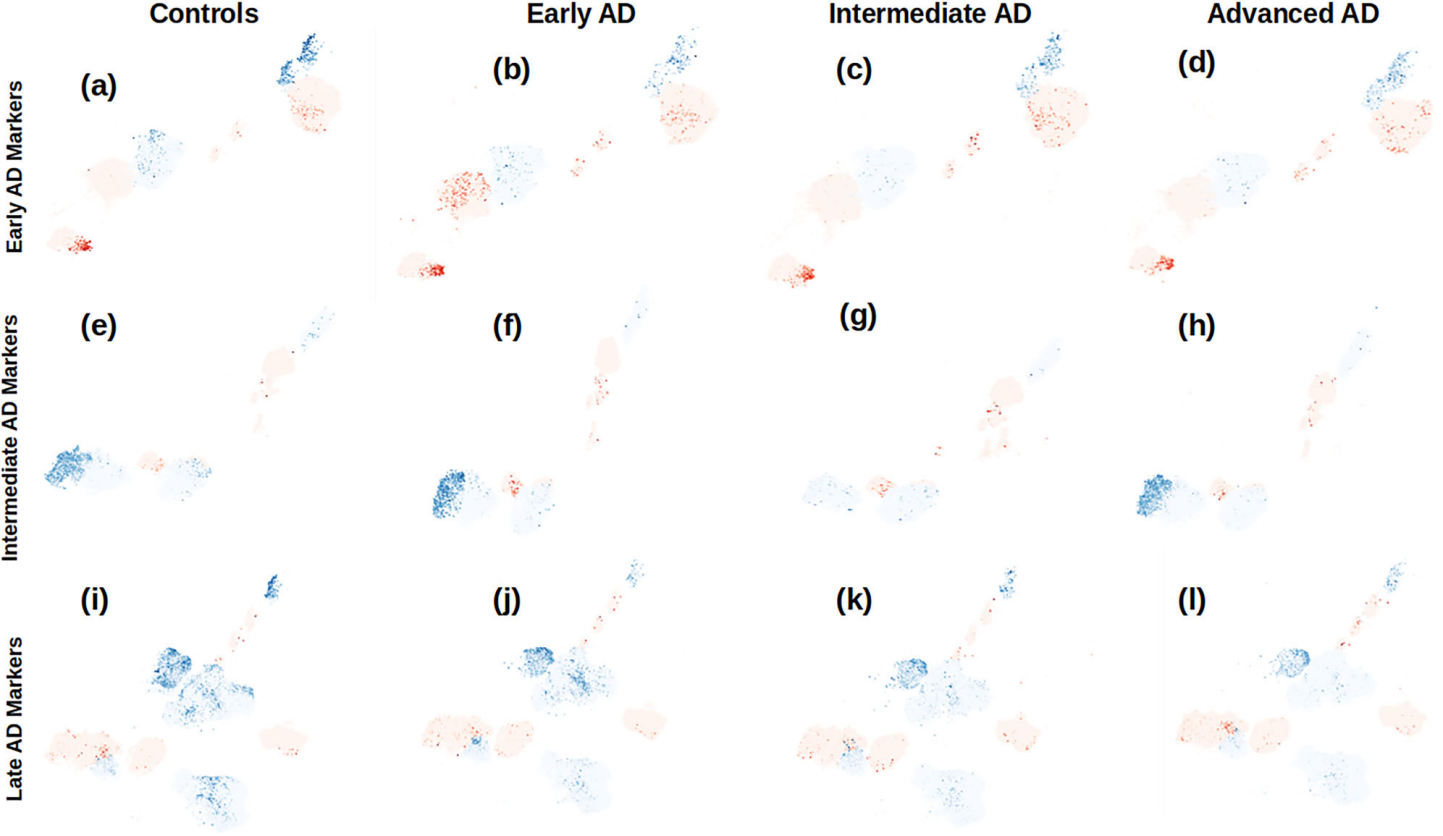
Marker genes for each AD stage are projected onto the UMAP embedding of the control samples’ cells and the cells of each AD stage. The genes marked in red are upregulated in the respective AD stage, and the ones marked in blue are found to be downregulated: ***a–d*** show the embedding of gene markers of early AD; ***e–h*** show the markers of intermediate AD; and ***i–l*** show the gene markers of advanced AD.

To track the progress of marker genes in all stages of AD, we show the major up- and downregulated genes from each stage along all the disease stages together with the control samples’ cells. Table 1 shows the projected genes from each stage, and Figure 5 shows the UMAP embedding of the respective genes in the cell type in which they have the maximum significant log-fold change in the respective AD stage. In all the three AD stages, we found known AD genes that are downregulated in all the stages and continue to downregulate through the stages, like *CRH* and *VGF* which are downregulated in both GABAergic and glutamatergic cells, *SST* in various GABAergic cells including Sst cells (except in early AD where it is also downregulated in glutamatergic L6 IT Car3 cells), and *ST8SIA2*, in L6 IT Car3 cells in all three stages. The downregulation of *CRH* in the cerebral cortex has been implicated in the pathogenesis of AD (Pedersen et al., 2001; Vandael and Gounko, 2019); *VGF*, a nerve growth factor-inducible gene, has been widely studied and established as a protector against AD; and its downregulation has been associated in mice models with neuronal activity, neural progenitor proliferation, memory formation, and depression-like behavior (Beckmann et al., 2020). Mathys et al. (2023) found VGF to be positively correlated with cognitive decline. Neuropeptide *SST* and its downregulation have been associated with formation of Aβ plaques via upregulation of neprilysin-catalyzed proteolytic degradation (P. Davies et al., 1980; Watamura et al., 2021). Persistent downregulation of *ST8SIA2* through the AD stages is already discussed in the section of DEGs in intermediate AD. We found GFAP (glial fibrillary acidic protein), a known AD biomarker (Gabitto et al., 2023; K. Y. Kim et al., 2023), to be upregulated through the AD stages, and maximally in glutamatergic L5/6 NP cells. Mathys et al. (2019) also found it upregulated in excitatory neurons and astrocytes (we also found it significantly upregulated in non-neuronal endothelial cells but only in intermediate AD); we found the upregulation of *CYSLTR2* (cysteinyl leukotriene receptor 2) to increase through intermediate and advanced AD in excitatory neuronal cells (Michael et al., 2021) and *LGR6* that continues to be upregulated through the stages in excitatory L6b cells. Both *GFAP* and *CYSLTR2* are known to increase neuroinflammation, *GFAP* via interactions with inflammatory cytokines (Azzolini et al., 2022) and *CYSLTR2* by virtue of being a lipid mediator of inflammation. Neuroinflammation suggests dysregulation of inflammation–resolution, and dysregulation of *LGR6* impedes macrophage phagocytosis, required for resolution of inflammation (Serhan et al., 2009; Emre et al., 2022).

**Table 1.** The dysregulated genes projected in UMAP embedding of Figure 5

	Early AD	Intermediate AD	Advanced AD
Upregulated genes	*ADGRG7*, *CSF3*, *CXCL1 CXCL2*, *CXCL3*, *EDA2R*, *GFAP*, *GPER1*, *HTR2B*, *MT1A*, *NOX4*, *RAB37*, *RAB38*, *ROS1*, *SELE*, *SERPINA3*, *SOCS3*, *TCIM*, *TNFRSF6B*, *VCAN*	*CSF3*, *CXCL10*, *C7*, *F2RL3*, *GFAP*, *HAMP*, *HBA1*, *MAL*, *MT1A*, *NOX4*, *NPAS4*, *QRFPR*, *SCIN*, *SLC26A2*, *SMO*, *STC1*, *TFPI2*, *TNFAIP8L3*, *TNFRSF6B*, *ZNF676*	*APOC2*, *CASR*, *CCL2*, *CYSLTR2*, *C1QC*, *C1QTNF1*, *DEFB1*, *ELOVL2*, *FMO3*, *GFAP*, *HBA1*, *HLA-DRA*, *IFNLR1*, *INSYN2B*, *LGR6*, *PRKCD*, *RGR*, *SOCS3*, *SULF1*, *TMED1*, *TXNIP*
Downregulated genes	*CDKN2A*, *DCDC1*, *DNAJB1*, *HSPA1A*, *HSPA1B*, *HSPA6*, *HSPB1*, *HSPH1*, *SST*, *ST8SIA2*	*CCDC88B*, *CXCR4*, *DCDC1*, *DKK2*, *DNAJB1*, *HSPA1A*, *HSPA1B*, *HSPA6*, *HSPB1*, *INHBA*, *ITK*, *KANK4*, *NPFFR2*, *SLCO1B1*, *SNCAIP*, *ST8SIA2*, *TRPC3*, *VGF*	*CRH*, *DNAJB1*, *GPRC5A*, *HSPA1A*, *HSPA1B*, *HSPA6*, *INHBA*, *NPTX2*, *PAPPA*, *PDE10A*, *SPRY4*, *SST*, *ST8SIA2*, *TRPC3*, *VGF*

Apart from protein coding GPCR family *ADGRG7* and the Rab genes, *RAB37* and *RAB38*, that are exclusively implicated in early AD, as mentioned earlier, we found several genes in vascular cell types that are significantly upregulated in early AD and are either not differentially expressed or slightly downregulated (statistically significant but with smaller log-fold change) in advanced AD. One such example is TNF (tumor necrosis factor) family receptor *EDA2R* (ectodysplasin A2 receptor) that is proinflammatory and has been found to be associated with proinflammation of tau protein (Duan et al., 2021) and was also found to be the strongest protein biomarker of cognitive ability in the plasma of a cohort of aged individuals (Harris et al., 2020); we found it to be significantly upregulated in endothelial cells of early AD samples. Similarly, *GPER1* (G-protein–coupled estrogen receptor 1) was exclusively upregulated in early AD, which is consistent with the findings of Oveisgharan et al. (2023), who have found the DNA methylation of it to be associated with core AD pathology like cognitive decline and tau-tangle density in females, which is also consistent with the SEA-AD data which have more female subjects in general, in addition to the observations in Sato et al. (2023) where it is touted to be associated with synaptic efficacy. *NOX4* (NADPH oxidase 4) has been shown by Park et al. (2021) to promote ferroptosis in non-neuronal cells via oxidative stress-induced lipid peroxidation by dysregulation of metabolism of mitochondria; we found it to be upregulated in all AD stages, but its log-fold change is maximum in endothelial cells of early AD; the respective endothelial cells’ log-fold change in early through advanced AD is 2.41, 2.23, and 1.75, respectively. Another interesting find, *TCIM* (transcriptional and immune response regulator), that is significantly upregulated in early and intermediate AD (respective log-fold change, 2.01 and 1.22) in vascular endothelial cells has been found to mediate inflammatory responses in endothelial cells via NF-kB signaling (T. Liu et al., 2017; J. Kim et al., 2009); we also found it significantly downregulated in VLMC in advanced AD (log-fold change, −1.03).

We also observed a third kind of trajectory of DEGs, in which the dysregulation increases from early to intermediate AD and then plummets in advanced AD. One such gene is *STC1* that in a study on humans was shown to have been overexpressed in the cerebrospinal fluid of patients with other dementias and ultimately had inconclusive results for AD dementia (Shahim et al., 2016), and on the contrary, in a study in mice, its overexpression was found to improve cognition and neuroinflammation (P. Wang et al., 2021). We found *STC1* significantly upregulated in the vascular cell types of early and intermediate AD (log-fold change of 0.96 and 2.06, respectively) and downregulated in advanced AD (log-fold change of −0.5 in endothelial cells). Additionally, we found that *SMO* (smoothened) gene, which encodes a GPCR that participates in sonic hedgehog (SHH) signaling pathway (Twigg et al., 2016; Jamieson et al., 2020), is exclusively differentially expressed (upregulated) in the inhibitory Sst Chodl cells of intermediate AD (log-fold change, 2.42). SMO-SHH signaling pathway has been investigated for therapeutic interventions to treat neurocomplications of AD (Prajapati et al., 2023), and W. Ma et al. (2018) also found it to modulate Aβ toxicity in neuroinflammation and cognition in AD. Lastly, similar to *SMO*, we found *F2RL3*, coagulation factor II (thrombin) receptor-like 3 (also encodes for a GPCR family), exclusively upregulated only in the endothelial cells of intermediate AD (log-fold change, 2.5). It has been known to induce apoptosis in AD (G. Wang et al., 2012), regulation of beta secretase cleavage of amyloid precursor protein (Xie and Guo, 2005), and inducement of hyperphosphorylation of tau proteins (Suo et al., 2003).

### Pathway analysis by GSEA in various AD stages

**Figure 6.**
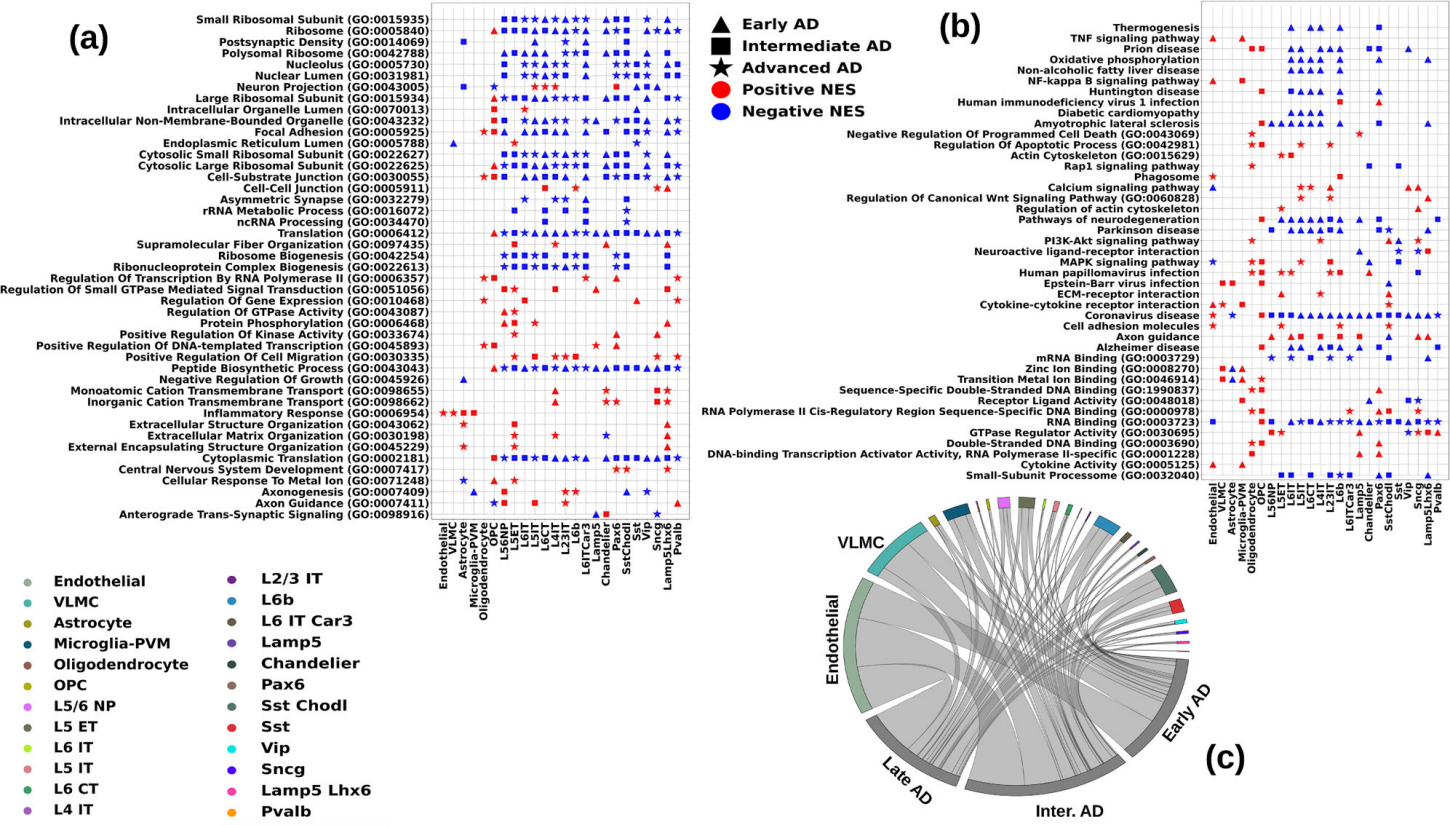
Summary of GSEA pathways in the three AD stages: triangle, square, and star, respectively, represent early, intermediate, and advanced AD, and the pathways with positive and negative normalized enrichment scores are represented by colors red and blue, respectively. ***a***, ***b***, Most significant KEGG and GO pathways in various AD stages in the cell types with significant dysregulation, indicating the AD stage in which the dysregulation (top, red; bottom, blue) is maximum. ***c***, Circos plot depicting the number of protein coding genes in the leading-edge genes of the significant (filtered at NOM *p* value of 0.01) GSEA pathways in each cell type in the three AD stages. All the significant pathways filtered at NOM *p* value of 0.01 are in Extended Data Figures 6-1–6-3 for early, intermediate, and advanced AD, respectively. The respective unfiltered results can be found in Extended Data Tables 6-1–6-3.

We performed pathway analysis by GSEA, and the results revealed various cell-type–specific gene sets dysregulated either in specific AD stages or common to multiple AD stages. We show in Figure 6*a*,*b* the significantly perturbed pathways in the three AD stages in each cell type, highlighting the AD stage with maximum enrichment. In Figure 6*c*, we show the number of genes driving these pathways in each cell type, in each AD stage. Cell-type–specific evidences of neurodegeneration such as downregulation of ribosomal proteins and translation-related genes were more prevalent and had earlier onset in L5 ET cells and were seen also in Pax6, Sst, Chandelier, and Sncg cells. Other pathways indicative of neurodegeneration (gene sets, Pathways of neurodegeneration, Parkinson disease, Huntington disease, Amyotrophic lateral sclerosis, Alzheimer's disease) were also downregulated in L5 ET, L5 IT, L6 CT, L4 IT, L6b, L6 ITCar3, Chandelier, Sst, Vip, and Lamp5Lhx6 neurons. Of these neuronal populations, the greatest neurodegenerative-like transcriptional changes identified by these pathways were in L5 ET and Sst Chodl neurons through the AD stages.

10.1523/ENEURO.0118-24.2024.f6-1 Figure 6-1 Significantly enriched GSEA pathways in early AD, filtered at nominal p-value 0.01. Download Figure 6-1, TIF file.

10.1523/ENEURO.0118-24.2024.f6-2 Figure 6-2 Significantly enriched GSEA pathways in intermediate AD, filtered at nominal p-value 0.01. Download Figure 6-2, TIF file.

10.1523/ENEURO.0118-24.2024.f6-3 Figure 6-3 Significantly enriched GSEA pathways in advanced AD, filtered at nominal p-value 0.01. Download Figure 6-3, TIF file.

10.1523/ENEURO.0118-24.2024.t6-1 Table 6-1 GSEA pathways in early AD. Download Table 6-1, XLS file.

10.1523/ENEURO.0118-24.2024.t6-2 Table 6-2 GSEA pathways in intermediate AD. Download Table 6-2, XLS file.

10.1523/ENEURO.0118-24.2024.t6-3 Table 6-3 GSEA pathways in advanced AD. Download Table 6-3, XLS file.

Inflammatory responses were activated in endothelial, VLMC, astrocytes, and microglia–PVM primarily in intermediate and advanced AD (gene sets, Cytokine-cytokine receptor interactions; Positive regulations of cytokine production; Inflammatory response). Extended Data Figures 6-1–6-3 depict the pathways with nominal *p* value of <0.01). We found that adenylate cyclase-activating GPCR signaling pathway is dysregulated in GABAergic Sst Chodl cells in early AD, and we found one of the lead genes, *ADGRG7*, to be a marker of early AD. Likewise, KEGG's cAMP signaling pathway is a significant endothelial marker pathway in intermediate AD. Exclusively and negatively enriched pathways in advanced AD include regulation of angiogenesis and regulation of vasculature development in endothelial cells, indicative of vascular disintegration and positive enrichment of regulation of autophagy in L5 ET cells. For the complete GSEA results on all AD stages, please see Extended Data Tables 6-1–6-3 with no filtration and Extended Data Figures 6-1–6-3 for the pathways with nominal *p* value of <0.01.

We found synaptic signaling and neuron projection pathways like anterograde transsynaptic signaling, chemical synaptic transmission, postsynaptic density, neuron projection guidance, neuron projection, and asymmetric synapse to be significantly negatively enriched in the inhibitory cell types and L6 IT Car3 excitatory cells in all AD stages, which is consistent with the findings of Mathys et al. (2023). As expected, we found cell-death–related apoptotic processes more dysregulated in intermediate and advanced AD in various neuronal and glial (oligodendrocytes and OPC) cells, also consistent with Mathys et al. (2023).

We found pathways indicative of microglia activation to be associated with all the AD stages, namely, cytokine-cytokine receptor interaction, inflammatory response, cytokine receptor, and cytokine-mediated signaling pathway. Sun et al. (2023b) classified and analyzed multiple microglial states in AD; MG10, the inflammatory microglial state identified by them to induce maximum inflammation, is enriched in GO pathways that parallel the present findings. Additionally, Sun et al. (2023b) identified MG11 as an antiviral microglial state, because of its enrichment in defense response to virus; we found in intermediate AD microglia–PVM cells enriched in the KEGG pathway of viral protein interaction with cytokine and cytokine receptors (Extended Data Fig. 6-2). We found *PDGFRB* to be exclusively downregulated in intermediate AD (log-fold change, −1.3) in vascular endothelial cells. With the dysregulation of pathways related to the cerebral vasculature of AD, described in Sun et al. (2023a), the authors identify *PDGFRB* as a significant pericyte cells’ marker that indicates blood–brain barrier disintegration.

### Intersection of differential expression and pathway analysis in AD stages and AUD

**Figure 7.**
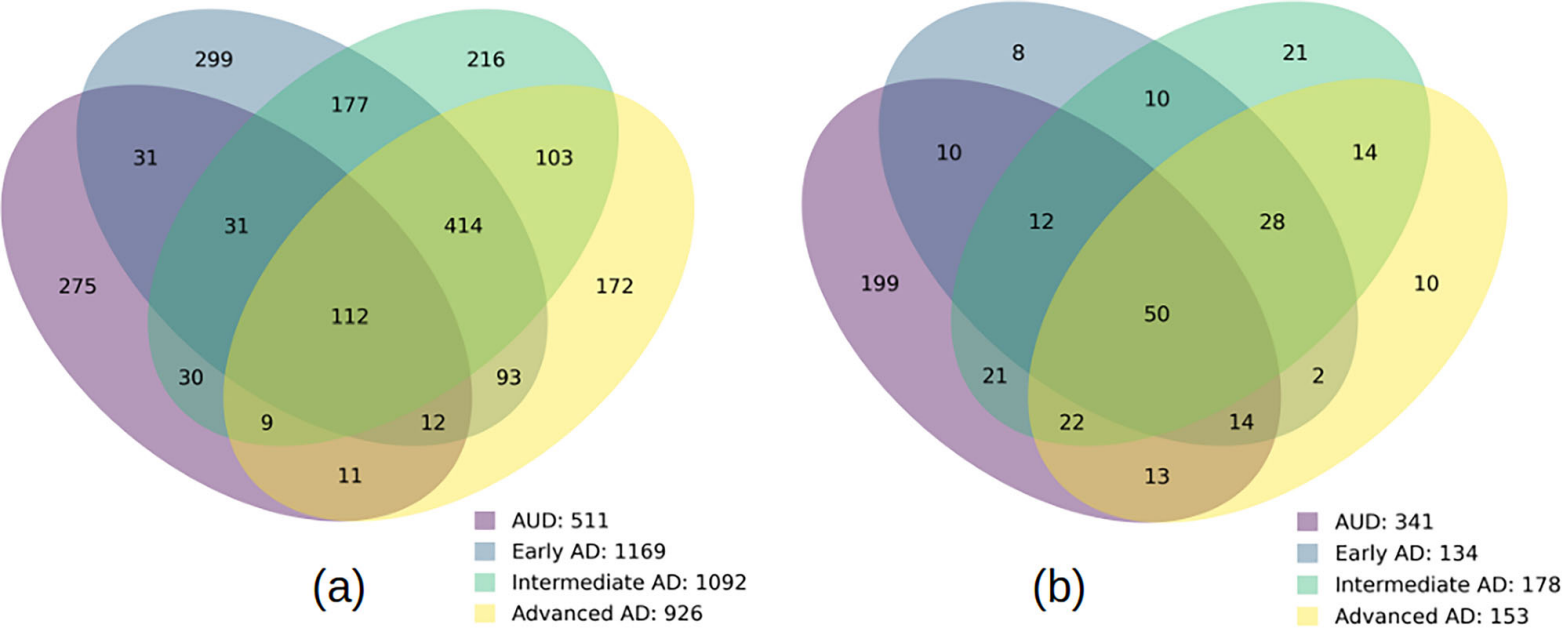
Venn diagrams representing the overlap between the three AD stages and AUD. ***a***, Intersection of significant (FDR *p* value of <0.05) protein coding DEGs, (***b***) intersection of significant (nominal *p* value of <0.01) GSEA pathways.

**Figure 8.**
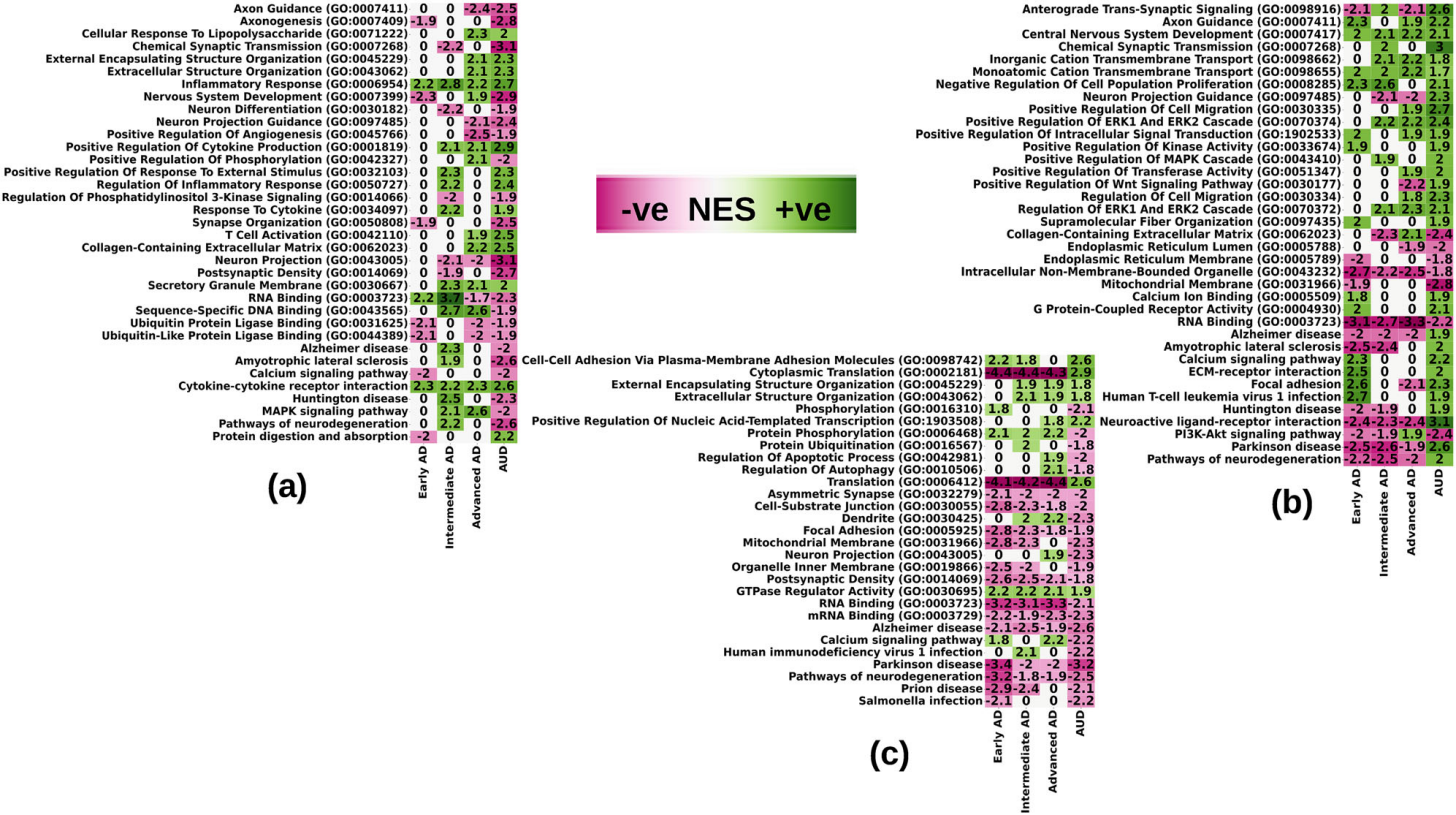
Respective annotated NES of some overlapping GSEA pathways in AUD and the three AD stages in the three cell-type families: (***a***) non-neuronal cells, (***b***) inhibitory neurons, (***c***) excitatory neurons.

We used the AUD dataset of Brenner et al. (2020) to compute the cell-type–specific DEGs and the perturbed GSEA pathways of the same cell-type families for AUD, as done for the three AD stages. In Figure 7*a*, we show the extent of overlap of the protein coding DEGs in AUD and the three AD stages. Figure 7*b* shows the same for the significant (filtered at nominal *p* value of 0.01) GSEA pathways. Figure 8*a–c* shows the heatmap of the significant overlapping GSEA pathways in AUD, with nominal *p* value threshold of 0.01 in at least one AD stage in the same family of cell types. The cell type chosen to display for each pathway is the one that has the highest NES in AUD. The zeros in all the heatmaps of Figure 8 indicate that either there is no enrichment, or it does not pass the threshold of the nominal *p* value. Next, we present significant GSEA pathways dysregulated in both AUD and AD in the three cell-type families.

#### Non-neuronal cells

Among the common pathways with increased expression in both AUD and AD are pathways relevant to inflammation; we found these pathways enriched in microglia and endothelial cells in AUD as well, as can be seen in Figure 8*a*. Likewise, angiogenesis in vascular cells and microglial cell activation-related pathways are similarly dysregulated in non-neuronal cells in AD and in AUD, shown in Figure 8*a*. For example, we found the T-cell activation pathway to be upregulated in Microglia in advanced AD, and its dysregulation in AUD (also from microglial cells) can be seen in Figure 8*a*. Other pathways indicative of inflammation, like cytokine production, inflammatory response, and cytokine-cytokine receptor interaction, are also similarly upregulated in AUD, as in the various AD stages.

#### GABAergic/inhibitory neurons

We found calcium signaling, axon guidance, and synaptic signaling-related pathways to be enriched in inhibitory/GABAergic neurons, and they are significant markers of all AD stages. These pathways were also enriched in AUD inhibitory neurons (Fig. 8*b*). Another inhibitory neurons’ signifier pathway in AD, PI3K-Akt signaling pathway, is also significantly dysregulated in inhibitory neurons in AUD (Fig. 8*b*). Likewise, we found significant downregulation in pathways termed, intracellular nonmembrane-bound organelle, mitochondrial membrane, and endoplasmic membrane and positive regulation of intracellular signal transduction, suggesting a similar disruption of intracellular mechanisms of inhibitory neurons in AD and AUD. We also found extracellular signal-regulated kinase (ERK) pathways upregulated in both AD and AUD.

#### Glutamatergic/excitatory neurons

In excitatory neurons, Figure 8*c*, we finally see the dysregulation of pathways indicative of neurodegeneration, namely, pathways of neurodegeneration, Alzheimer's disease, and Parkinson's disease, dysregulated in the same direction (downregulated) which were dysregulated in opposite directions in non-neuronal cells and inhibitory neurons in AD and AUD. Transcription and translation-related pathways like nucleic acid-assisted transcription and mRNA and RNA binding are also enriched similarly in AUD as in the various AD stages. Pathways indicative of synaptic disintegration, like asymmetric synapse and postsynaptic density are also significantly downregulated in both AD and AUD, which indicates additive synaptic disintegration in both diseases. Similar to inhibitory neurons, we observed significant dysregulation in the pathways termed organelle inner membrane, mitochondrial membrane, and nuclear membrane, suggesting a similar disruption of intracellular mechanisms of excitatory neurons as well in AD and AUD. A significant find in excitatory neurons is the dysregulation of tubulin binding in AUD (not shown in the figure because the pathway did not cross the significance threshold in AD in the SEA-AD dataset). Tubulin binding has been found to be significantly lower in AD patients due to deposition of hyperphosphorylated tau proteins that have low tubulin affinity which causes microtubule destabilization, ultimately leading to compromised axonal and synaptic integrity (Zhang et al., 2015; Fernandez-Valenzuela et al., 2020; Santiago-Mujika et al., 2021).

The significant overlap of DEGs and GSEA pathways between AUD and the various AD stages hints at a deeper transcriptional relationship between AUD and the AD pathology. This led us to investigate the footprints of AUD gene markers in the three AD stages.

### Dysregulated AUD GSEA in AD stages

**Figure 9.**
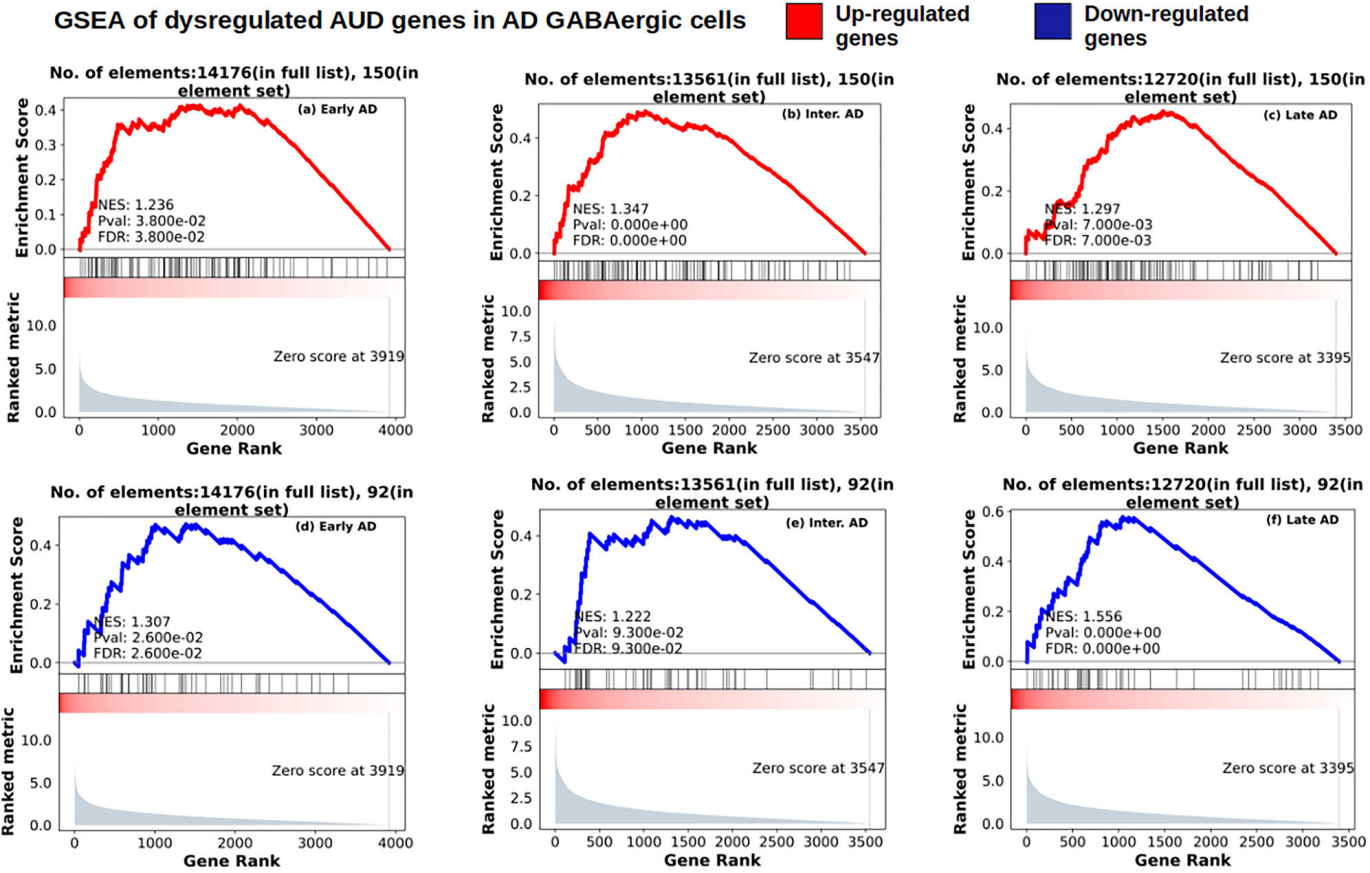
GSEA plots of upregulated (upper row, red) and downregulated (lower row, blue) AUD marker genes in GABAergic cells as a set to query the differential expression signature of the various AD stages in the GABAergic cells: (***a***, ***d***) Early AD; (***b***, ***e***) Intermediate AD; (***c***, ***f***) Advanced AD.

We used cell-type–specific marker AUD genes as gene sets to query the corresponding cell type and cell-type family–specific differential expression signature of the AD stages. This exhaustive search led to the GABAergic/inhibitory cell-type family of AUD marker genes having majorly significant enrichment in the differential expression signature of intermediate and advanced AD. The upregulated AUD markers have maximum significant enrichment in intermediate AD, as shown in Figure 9*a–c*, while the downregulated AUD markers, Figure 9*d–f*, have maximally significant enrichment in advanced AD.

**Figure 10.**
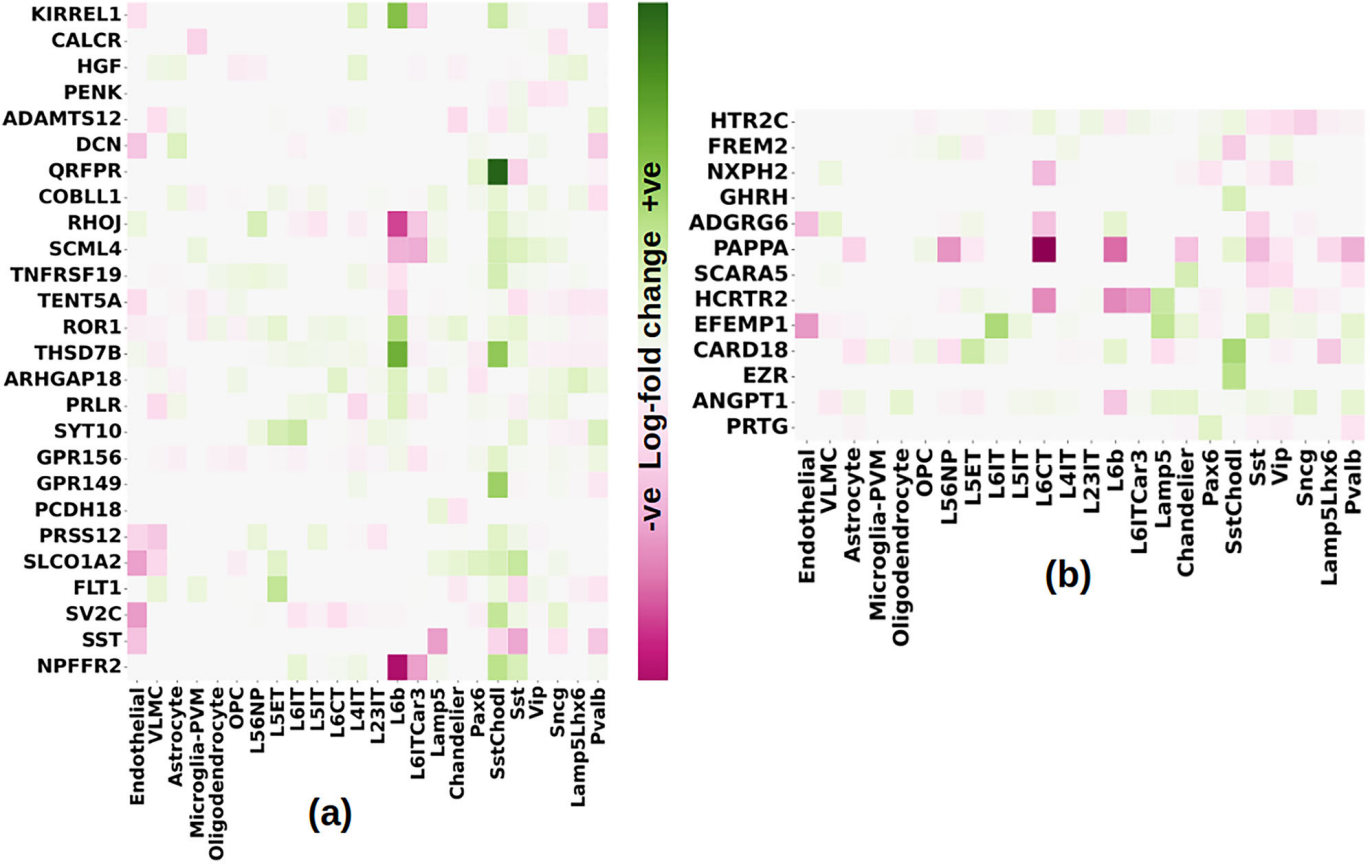
Cell-type–specific distribution of log-fold change of the DEGs in the leading edge of the marker AUD gene sets in intermediate and advanced AD: (***a***) upregulated AUD genes in the leading edge of differential expression signature of intermediate AD that are also significantly differentially expressed in intermediate AD; (***b***) downregulated AUD genes in the leading edge of differential expression signature of advanced AD that are also significantly differentially expressed in advanced AD.

Some of the genes that drive the leading edge of the corresponding enrichment in intermediate and advanced AD are also significantly differentially expressed in the corresponding AD stages. In Figure 10, *a* and *b*, we show the heatmap in various cell-types of these overlapping genes in intermediate and advanced AD respectively. Significant upregulation can be seen in Sst Chodl GABAergic cells in intermediate AD. One significant result is *QRFPR* (pyroglutamylated RFamide peptide receptor) or GPCR 103. J. Davies et al. (2015) show that QRFP treatment modulates tens of genes that dysregulate *MAPK* and PI3K-Akt signaling pathways which we found dysregulated significantly in all AD stages (Fig. 6*b*). In advanced AD too, throughout GABAergic cell types, downregulation of these leading-edge genes that are also differentially expressed in advanced AD can be seen. Cell-type–specific modulations of the log-fold change can be seen in *EFEMP1*, *CARD18*, *SCARA5*, and *HCRTR2*. Interestingly, *PAPPA* (pregnancy-associated plasma protein-A) that is significantly downregulated in advanced AD (Figs. 4*c*, 10*b*) in inhibitory and excitatory neurons is known to cleave insulin growth factors (Bøtkjær et al., 2019), which contribute to hypoglycemia and have implicitly associated risk factors to Aβ protein dysregulation (Moin et al., 2021). Similarly, *HCRTR2*, one of the two hypocretin/orexin receptors, regulates sleep (Z. Ma et al., 2016), which in turn has been shown to be disrupted in AD patients (Fronczek et al., 2012; Scammell et al., 2012).

While the significant enrichment of significant DEGs of AUD in the differential expression signature of intermediate and advanced AD makes a strong indication toward AUD exacerbating AD, we wanted to further examine the gene networks associated with AUD and their regulation in the various stages of AD. We next present the results of the master regulator analysis (MRA) using “*corto*” (Mercatelli et al., 2020) for the transcription factors among the significantly differentially expressed AUD genes.

### Gene network perturbation of AUD markers in advanced AD

**Figure 11.**
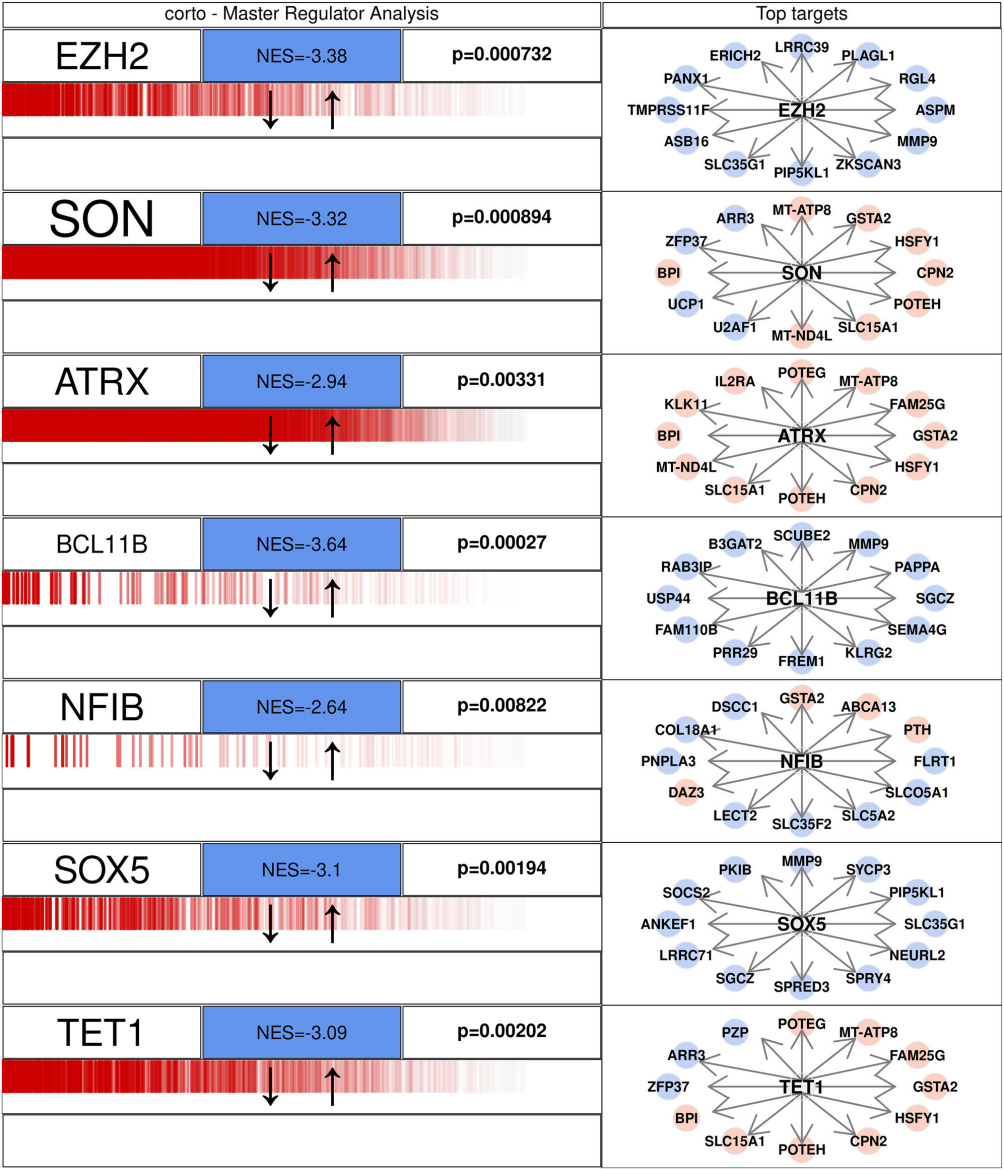
Master regulator analysis (MRA) of the significantly differentially expressed transcription factors in transcription profiles of AUD samples in advanced AD. Genes on the left are the selected transcription factors found dysregulated in AUD; their transcriptional signature from advanced AD is compared with controls. Extended Data Table 11-1 shows the entire networks of these genes. Additionally, Extended Data Figures 11-1 and 11-2 show the same for early and intermediate AD.

We used 52 transcription factors that are also significantly differentially expressed in AUD as centroids to create a correlation network of genes from the pseudobulked expression profiles of all three stages of AD. Then analyzing these networks in the control samples and the samples of each stage of AD separately through MRA as implemented in Mercatelli et al. (2020) resulted in significant correlation of the transcription factors’ and their corresponding target genes’ association in advanced AD (Fig. 11). The chosen transcription factors are the genes significantly dysregulated in AUD. The transcriptional signature of these transcription factors is then compared in control samples versus AD using MRA. In early and intermediate AD, the results are either statistically not significant or very weak, as can be seen in Extended Data Figures 11-1 and 11-2. Some of the transcription factors whose networks are strongly perturbed in both AUD and advanced AD are depicted in Figure 11: *EZH2* (enhancer of zeste 2 polycomb repressive complex 2 subunit), *SON*, *SOX5*, *BCL11B*, *NFIB*, *ATRX*, and *TET1*; the entire network of these transcription factors can be found in Extended Data Table 11-1. *EZH2* is a transcriptional repressor, mutations of which cause Weaver syndrome (Gibson et al., 2012), a rare genetic disorder characterized by intellectual disability. *SON* is a DNA and RNA binding protein, and its mutations cause Zttk (Zhu–Tokita–Takenouchi–Kim) syndrome, another severe multisystem developmental disorder characterized by intellectual disability and psychomotor development (Tokita et al., 2016). Similarly, mutations in *ATRX* (alpha-thalassemia/mental retardation, X-linked) cause the alpha-thalassemia X-linked intellectual disability syndrome, and acquired mutations have been observed in multiple types of cancers like gliomas (Stevenson, 2000); *SOX5* [sex determining region Y (SRY)-related high mobility group (HMG) box family of proteins] is also known for intellectual disabilities (Lamb et al., 2012; Schanze et al., 2013) and dysregulation of chondrogenesis (C-F. Liu and Lefebvre, 2015). *NFIB* (nuclear factor I/B) too is associated with intellectual disability and acts as a transcription activator of *GFAP* (Schanze et al., 2018), which is found to be significantly upregulated in all AD stages in this work and is also essential for proper brain development (Schanze et al., 2018). *TET1* (ten–eleven translocation methylcytosine dioxygenase 1) has *NPAS3* (neuronal Per-Arnt-Sim 3) as a target (Extended Data Table 6-2) that is associated with schizophrenia (Kamnasaran et al., 2003) essential for synaptic plasticity in turn affecting learning and retention. *SGCZ* (sarcoglycan zeta), found as a target of both *BCL11B* and *SOX5* (Fig. 11), is known to be associated with hallucinogen abuse (T. Wang et al., 2017). We found *SPOCK2* as a target of *ATRX* and *SPOCK3* as a target of *TET1* (Extended Data Table 6-2, a paralog of which, *SPOCK1*, was recently found to make region-specific blood–brain barrier permeable in neurodegenerative diseases in zebrafish and mice, making the brain more susceptible to infections (O’Brown et al., 2023). Lastly, better assimilation of dietary zinc has been shown to possess ameliorative effects in AD progression (Rivers-Auty et al., 2021; Hafez et al., 2023). The three master regulators from AUD transcription factors of advanced AD above, *BCL11B*, *ATRX*, and *TET1* are transcription factors for zinc finger-binding proteins as well. *BCL11B* encodes “C2H2” type domains (Eto et al., 2022); *ATRX* too responds to atypical chromatin signatures and binds to zinc finger protein encoders (Valle-García et al., 2016). *TET1* is majorly involved in DNA methylation and affects chromatin regulation and accessibility, which is supported by CXXC-type zinc finger proteins (Ono et al., 2002; Arroyo et al., 2022; Stolz et al., 2022).

10.1523/ENEURO.0118-24.2024.f11-1 Figure 11-1 Master Regulator Analysis of the significantly differentially expressed transcription factors in transcription profiles of AUD samples in early AD. Genes on the left, are the selected transcription factors found dysregulated in AUD, their transcriptional signature from early AD is compared with controls. Download Figure 11-1, TIF file.

10.1523/ENEURO.0118-24.2024.f11-2 Figure 11-2 Master Regulator Analysis of the significantly differentially expressed transcription factors in transcription profiles of AUD samples in intermediate AD. Genes on the left, are the selected transcription factors found dysregulated in AUD, their transcriptional signature from intermediate AD is compared with controls. Download Figure 11-2, TIF file.

10.1523/ENEURO.0118-24.2024.t11-1 Table 11-1 Networks of transcription factors depicted in Figure-11. Download Table 11-1, XLS file.

## Discussion

In this study, we profiled transcriptional changes associated with AD progression in the neocortex and compared them with transcriptional changes in AUD at the single-cell level. We observed a decline in the numbers of both glutamatergic and GABAergic neurons in AD. Among GABAergic neurons, Sst neurons were affected early in the disease. A decline in the relative abundance of GABAergic neurons and their AD susceptible subtype, Sst neurons, in the brains of cognitively compromised individuals was recently reported in another AD dataset (Mathys et al., 2023). Consens et al. (2022) reported significant reduction of both Sst and IT neurons in AD from multiple bulk and single-cell studies of AD and associated abundance of both with slower cognitive decline. We also observed a decline in glutamatergic neurons in both deep and superficial intratelencephalic (IT) and extratelencephalic (ET) excitatory neurons with progressing AD pathology. The decline of supragranular (L2/3) intratelencephalic-projecting excitatory neurons is in general agreement with findings in different AD datasets (Gabitto et al., 2023; Mathys et al., 2023). We also observed a decline of deep layer excitatory neurons that was associated with gene expression evidence of degenerative changes in these populations, as discussed below. In particular, in addition to intratelencephalic L5 IT, L2/3 IT, and L6 IT Car3, we also found extratelencephalic and near-projecting L5 ET and L5/6 NP neurons also significantly diminished in advanced AD (Fig. 3).

We identify several DEGs that are unique to an AD stage and also ones that are unique in terms of their diverse cell-type–specific expression in an AD stage. We also present various distinctively enriched gene set pathways that provide an insight into the cellular processes associated with AD progression in distinct cell types. For example, we found autophagy-related genes and pathways perturbed in early AD, which is consistent with previous findings (Uddin et al., 2018; Mathys et al., 2023), and a number of genes in vascular cell types that dysregulate transcription and are proinflammatory, like *TCIM* and *EDA2R*, suggesting these core AD marker pathologies begin in brain vasculature (Duan et al., 2021; J. Kim et al., 2009; T. Liu et al., 2017; Sun et al., 2023a). Likewise, in later stages neuroinflammation-related genes, including *GFAP* (Hill and Gammie, 2022; Gabitto et al., 2023; K. Y. Kim et al., 2023) and the stress peptide *CRH* (Pedersen et al., 2001; Koutmani et al., 2019; Vandael and Gounko, 2019; Hill and Gammie, 2022), are maximally dysregulated in excitatory neurons. *GFAP* expression in excitatory neurons in AD was also observed by Mathys et al. (2019). Similarly, synaptic signaling and cell-death–related pathways are also most significantly dysregulated in neuronal cell types in advanced AD, consistent with the dataset of Mathys et al. (2023).

Pathway analysis by GSEA revealed cell-type–specific evidence of neurodegeneration. Particularly affected were L5 ET, as well as Pax6, Sst Chodl, L6 CT, L4 IT, L6b, L6 ITCar3, Chandelier, Sncg, Sst, Vip, and Lamp5 Lhx6 neurons. Evidence of inflammatory responses was seen in non-neuronal cells throughout the AD stages. We found transcriptional evidence of increased glial apoptosis (oligodendrocyte and OPC; see Extended Data Figs. 6-2, 6-3) in intermediate and advanced AD. Plaque-associated microglia in AD can show apoptotic changes (Yang et al., 1998; Hansen et al., 2018). Increased apoptosis is shown by ineffective microglia such as TREM2-deficient microglia (Y. Wang et al., 2015). In another AD dataset, evidence of increased cell death in glial cells in late AD was also observed (Mathys et al., 2023).

The present work, to the best of our knowledge, is the only work that examines transcriptomic changes in both AD and AUD at the single-cell resolution in human datasets. We found apoptosis, autophagy, DNA methylation, inflammatory immune response related to HMG proteins, dysregulated neurotransmission caused by toxicity of excitatory neurons, cell death caused by BDNF receptors, and axonal damage, all dysregulated in either AD or both AD and AUD, consistent with other lines of evidence (Araujo et al., 2021; León et al., 2022; Pierson et al., 2024). We found *CRH*, as mentioned earlier, to be differentially regulated in AD but not AUD, similar to the report of M. Kapoor et al. (2021). Also, relevantly Ray et al. (2013) found its receptor *CRHR1* positively correlated with the onset and maintenance of alcoholism. We also found the *CFH* (complement factor H) gene upregulated in AUD but downregulated in early and intermediate AD. The complement system is implicated in the pathogenesis of AD, neuroinflammation, and neurotoxicity (Dalakas et al., 2020; Jun et al., 2022; Ramos et al., 2022). Lastly, we showed in the section of DEGs of early AD that *NOX4* is one major ferroptosis-related gene upregulated in all AD stages in endothelial cells; we also found it upregulated in AUD. Dysregulation in ferroptosis-related genes has been shown to be predictive of both AUD and AD (Tian et al., 2023). We next discuss the overlapping dysregulations in each cell-type family for both the diseases.

We identify a considerable number of genes and pathways that are similarly perturbed in AD and AUD and are common to all three AD stages. Among them, expression of pathways indicative of inflammatory responses and cytokine activation was increased at all stages of AD and in AUD in non-neuronal cells. In the microglial dynamics of AD in Sun et al. (2023b), the proinflammatory MG10 microglial state aligns with our findings of enriched pathways associated with intermediate and advanced AD, along with AUD, including inflammatory response and cytokine signaling. Additionally, dysregulation of protein phosphorylation-related pathways dysregulated in non-neuronal cells with the advent of any neurodegenerative disease was also found in both AD and AUD; we also found T-cell activation pathway to be dysregulated in microglia in advanced AD and AUD (Fig. 8*a*). Consistently, Mathys et al. (2023) observed that cell activation pathways in microglia are among the most significant perturbations in late changes associated with AD.

The blood–brain barrier in neurodegenerative diseases has been extensively studied (Nation et al., 2019; Montagne et al., 2020; O’Brown et al., 2023; Sun et al., 2023a). We found *PDGFRB* to be exclusively downregulated in intermediate AD (log-fold change, −1.3) in vascular endothelial cells. *PDGFRB* is upregulated after brain injury, and its depletion results in blood–brain barrier damage; Sun et al. (2023a) highlighted *PDGFRB*, platelet-derived growth factor-BB, as a significant marker for pericyte cells in AD-related vascular changes (Sun et al., 2023a), consistent with our finding of its exclusive downregulation in endothelial cells of intermediate AD. Its signaling pathway, PDGF-BB:PDGFRβ and general pericyte loss in AD, is also well documented (Halliday et al., 2016; A. Kapoor et al., 2022; Smyth et al., 2022). Smyth et al. (2022) also show that PDGF-BB signaling is mediated by ERK and Akt pathways, which we have found to be dysregulated in multiple cell types including downregulation in VLMCs in intermediate AD (Extended Data Fig. 6-2, Fig. 8*b*). In addition to this, *SPOCK2* and *SPOCK3* [SPARC (osteonectin), cwcv- and kazal-like domains proteoglycan], found by MRA to be targets of *ATRX* and *TET1*, respectively, are transcription factors dysregulated in AUD and are a part of the SPARC (secreted protein acidic and rich in cysteine) family of proteins that regulate ECM signaling pathways that can be seen dysregulated in both AD and AUD in Extended Data Figures 6-1–6-3 and also Figure 8, and *SPOCK1* has been implicated in AD (O’Brown et al., 2023) via participation in the disintegration of blood–brain barrier. It has also been speculated by Ye et al. (2020) to be prompted by PDGF-BB via the activation of PI3K/Akt/FoxM1 signaling pathway to ultimately also affect ECM. Moreover, we also found *SCUBE2* (signal peptide, cubulin domain, epidermal growth factor-like 2) with MRA as a target of *BCL11B* (Fig. 11); it has been shown to promote blood–brain barrier disintegration via disruption of SHH pathway (Brilha et al., 2017; Shi et al., 2023), which in turn modulates Aβ toxicity in neuroinflammation and cognition in AD (W. Ma et al., 2018).

In excitatory neurons, we found multiple AUD perturbed pathways, including pathways associated with neurodegeneration and synaptic dysfunctionality, among others, that were similarly associated with multiple AD stages (Fig. 8*c*). The nuclear lumen, defined as the volume enclosed by the inner membrane of the nucleus, known to impair the nuclear waste elimination in AD (Frost, 2016), was found to be downregulated in excitatory and inhibitory neurons in all AD stages (Fig. 6*a*). In AUD we found a similar pathway, nuclear membrane, downregulated in AUD neurons as well, indicating that AD and AUD may produce additive nuclear damage. Common dysregulation of the nuclear lumen/membrane, synaptic asymmetry, postsynaptic density, synaptic signaling, and pathways of neurodegeneration in both excitatory and inhibitory neurons suggests that AD and AUD may converge in causing nuclear and synaptic damage, hallmarks of AD (Frost, 2016; Wu et al., 2021; Mathys et al., 2023). Moreover, we find that dysregulated AUD genes in inhibitory neurons are enriched in the differential expression signature of intermediate and advanced AD (Fig. 9), indicating that genetic network perturbation in AUD can exacerbate AD progression. Dysregulation of genes, in both AD and AUD, included *QRFPR* GPCR103, the neuropeptide QRFP receptor, and *HCRTR2*, one of the two hypocretin/orexin receptors that regulate sleep, which is disrupted in AD patients (Fronczek et al., 2012; Scammell et al., 2012; J. Davies et al., 2015; Z. Ma et al., 2016). *HCRTR2* has been implicated in substance use disorders (K. T. Schmeichel et al., 2015; Aldridge et al., 2022; Chen et al., 2024), and QRFP has been shown to exert neuroprotective effects in AD via heterodimerization of *QRFPR* with orexin receptors (J. Davies et al., 2015).

Results of the master regulator analysis (MRA) further support that AUD has the potential to promote AD progression, by revealing a significantly positive regulation of the transcription factor gene markers of AUD in advanced AD. We identified seven genes, *SON*, *EZH2*, *SOX5*, *BCL11B*, *NFIB*, *ATRX*, and *TET1*, some of which are known markers of intellectual disability (Stevenson, 2000; Gibson et al., 2012; Lamb et al., 2012; Schanze et al., 2013, 2018; Tokita et al., 2016) or target genes that are markers of neuroinflammation such as *GFAP* (Azzolini et al., 2022) or are associated with other neurodegenerative diseases like schizophrenia, AD itself, or substance abuse, like *SGCZ*, *SPOCK2*, *SPOCK3*, and *NPAS3* (Kamnasaran et al., 2003; O’Brown et al., 2023; T. Wang et al., 2017). Lastly, Mathys et al. (2023) also found *ATRX*, a major chromatin regulator involved in the DNA damage response, to be differentially regulated in late AD.

In this work, we covered the comprehensive pathology of AD to find the genetic changes that drive its progression and juxtapose the resulting perturbations with AUD. We found several cell-type–specific genes and pathways that are at the forefront of both the diseases, providing support that AUD can promote or accelerate AD progression. A limitation of the present study is the small sample size of the AUD dataset. Larger studies will be needed to better understand the contribution of excessive alcohol intake to the pathogenesis and progression of AD and to adequately represent all possible variants of central nervous system diseases.

## Synthesis

Reviewing Editor: Leandro Vendruscolo, National Institute on Drug Abuse Decisions are customarily a result of the Reviewing Editor and the peer reviewers coming together and discussing their recommendations until a consensus is reached. When revisions are invited, a fact-based synthesis statement explaining their decision and outlining what is needed to prepare a revision will be listed below. The following reviewer(s) agreed to reveal their identity: Katherine Savell, Caio Mazucanti.

Reviewer 1:

This study investigates the transcriptional landscape of the middle temporal gyrus in humans with varying stages of Alzheimer's disease (AD) and prefontal cortical samples from humans with alcohol use disorder (AUD). This is an important area of study due to the prevalence and increases of both AD and AUD, and the potential interaction of these diseases.

I commend the authors for using publicly available datasets and generating additional analyses that complement the original studies and synthesize these datasets in a novel way to investigate the potential interaction of AD and AUD. A descriptive manuscript is fine for a resource like this, but I have suggestions and additional analysis outlined below that should be addressed before publication so the analyses can be most useful to the field:

1. One point of discussion should be that the SEA-AD data that is used in this study is from a preprint and has not gone under the peer review process. Since this is a double-blind review, I have no way of knowing if the authors are related to the SEA-AD project or not, but this should be taken into consideration. I note that I referenced the SEA-AD preprint to cross check analyses for duplication.

2. I recommend the authors clearly state within Figure 1 that this is a publicly available dataset (in addition to listing it in the figure legend). I note a discrepancy in the number of control or ‘No AD’ samples between this submission and the SEA-AD dataset (Figure 1c in present study, Figure 1b in SEA-AD).

3. In Figure 1, it would be nice to see the cell type clusters plotted by group.

4. For Figure 2, in addition to the raw cell counts, authors should provide the proportion of cells collected for a donor and plot individual data points for raw counts and proportion. The SEA-AD preprint indicates that the QC of advanced AD patients was lower, which I assume means they collected fewer cells per Advanced AD patient. The proportion calculation (# cells in cell type/ # cells collected from patient) will indicate if it's a decrease across all cell types or if a particular cell type is affected based on what was collected.

5. For Figures 3-5, it is confusing to show one volcano plot then a cell-type specific examples. I recommend summarizing DEG numbers in a table. Can the authors comment on if the DEGs are consistent across patients? Also, is this three pairwise comparisons (Ctrl vs early, ctrl vs. int., ctrl vs. advanced)? Could all of this be combined into one figure?

6. Can the authors discuss how lvm-DE handles biological replicates? Is it by number of cells total or cells from a patient (true biological replicate).

7. Figure 10 is very nice. Figures 7-9 seem redundant. Can they be moved to a supplemental table instead of a full figure?

8. The AUD dataset and comparison seems like an afterthought. Can the authors show the cell types, cell type counts/ proportions, and DEGs for this dataset too? I understand that the data might look slightly different than in Brenner et al due to differences in cell type annotation, but it is important to show what you are comparing. Do they have similar cell types?

9. Overall, a lot of the text within the figures is small, inconsistently sized between and within figures, or stretched/squished. This makes reading and interpreting the figures difficult. I recommend carefully checking font sizes and scaling. Additionally, the panels being all slightly different sizes is distracting.

10. Is the analysis code uploaded to a repository? The authors should acknowledge how they will share this analysis.

Reviewer 2:

This manuscript offers a comprehensive and in-depth exploration of single-cell transcriptomic signatures at various stages of Alzheimer's Disease (AD) and Alcohol Use Disorder (AUD), yielding intriguing and significant discoveries. However, it is apparent that there are notable deficiencies in the graphical representations presented in this scientific manuscript.

The visual elements, intended to elucidate the data and findings, are substandard, unrefined, and lack visual appeal. The authors appear to have invested minimal effort in ensuring clarity or effectively utilizing the figures to support their points. Specifically, the figures demonstrate a lack of attention to detail and precision, resulting in a sense of sloppiness in their design and execution. This is evident in unclear labeling, inconsistent formatting, overcrowding of data points, and overall disorganized presentation. These factors collectively result in a visual presentation that does not meet the expected standards of clarity and professionalism in scientific communication.

Furthermore, certain figures are unintelligible or challenging to interpret due to their poor graphical quality. The visual complexity, combined with low resolution and inadequate design, hinders the effective communication of key data points, trends, and relationships. Consequently, it was challenging to extract meaningful insights or draw accurate conclusions from the visual representations provided.

The figure legends are also greatly inadequate. Firstly, they fail to provide sufficient descriptive information to help readers interpret the figures effectively. Secondly, figure legends should ideally provide context and highlight the key findings or insights that the figure is intended to convey. In this case, the figure legends do not effectively guide the reader towards understanding the significance of the data presented.

Given the critical role that figures play in conveying scientific information and enhancing the understanding of research results, it is imperative that these shortcomings are addressed. By improving the visual design, clarity, and overall quality of the figures, the manuscript can better fulfill its purpose of presenting and interpreting data in a clear, engaging, and informative manner for the benefit of the scientific community.
