# Supplementary figures and images for: Transcriptional Patterns in Stages of Alzheimer's Disease Are Cell-Type–Specific and Partially Converge with the Effects of Alcohol Use Disorder in Humans
Source: eNeuro. 2024 Oct 9;11(10):ENEURO.0118-24.2024. doi: 10.1523/ENEURO.0118-24.2024 (PMC11485264; doi:10.1523/ENEURO.0118-24.2024)

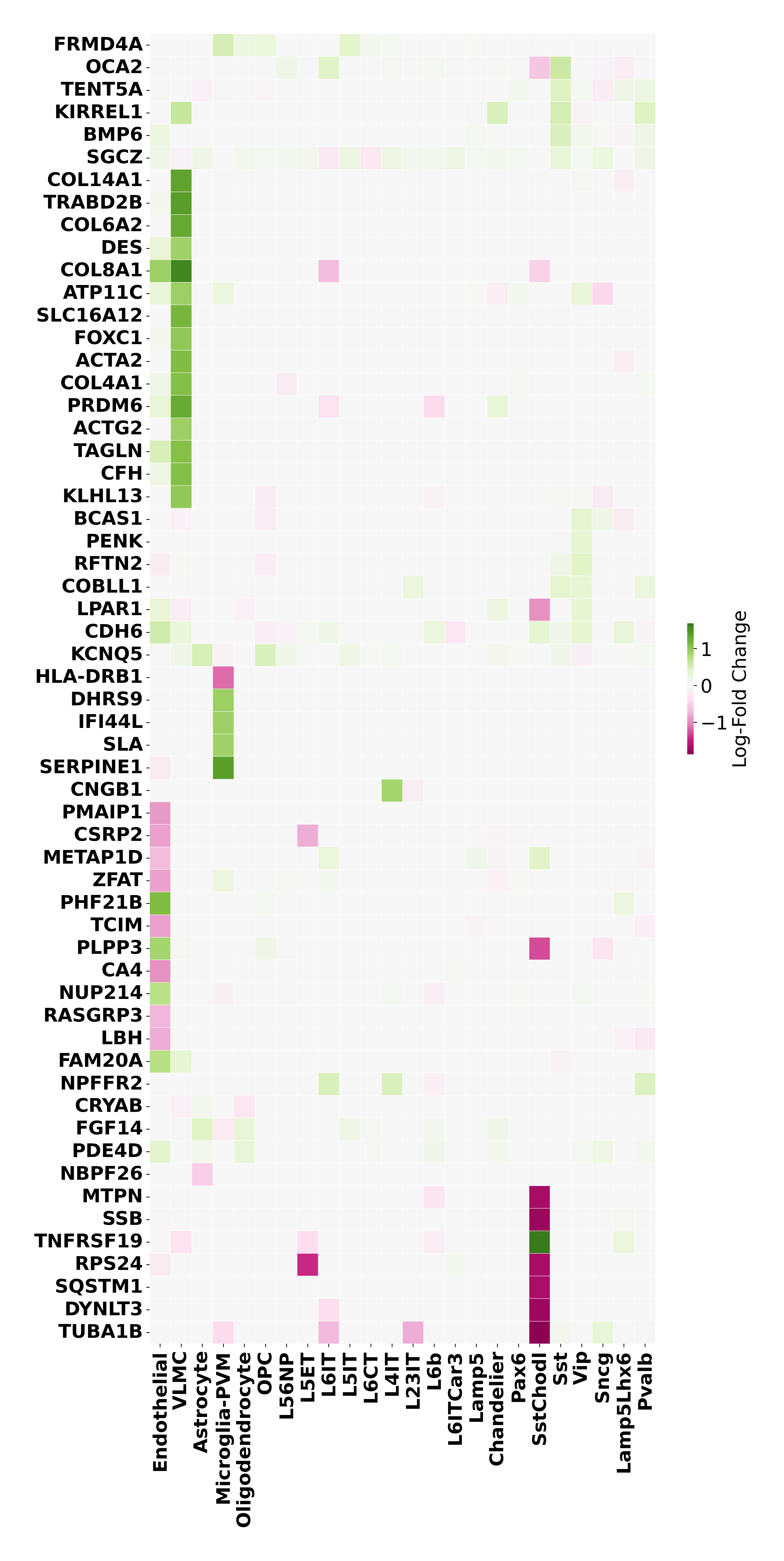

Supplement: Figure 2-1 — Most significant differentially expressed genes in the various cell types in the AUD data (Brenner et al., 2020). Download Figure 2-1, TIF file. [file eneuro-11-ENEURO.0118-24.2024-s001.tif]

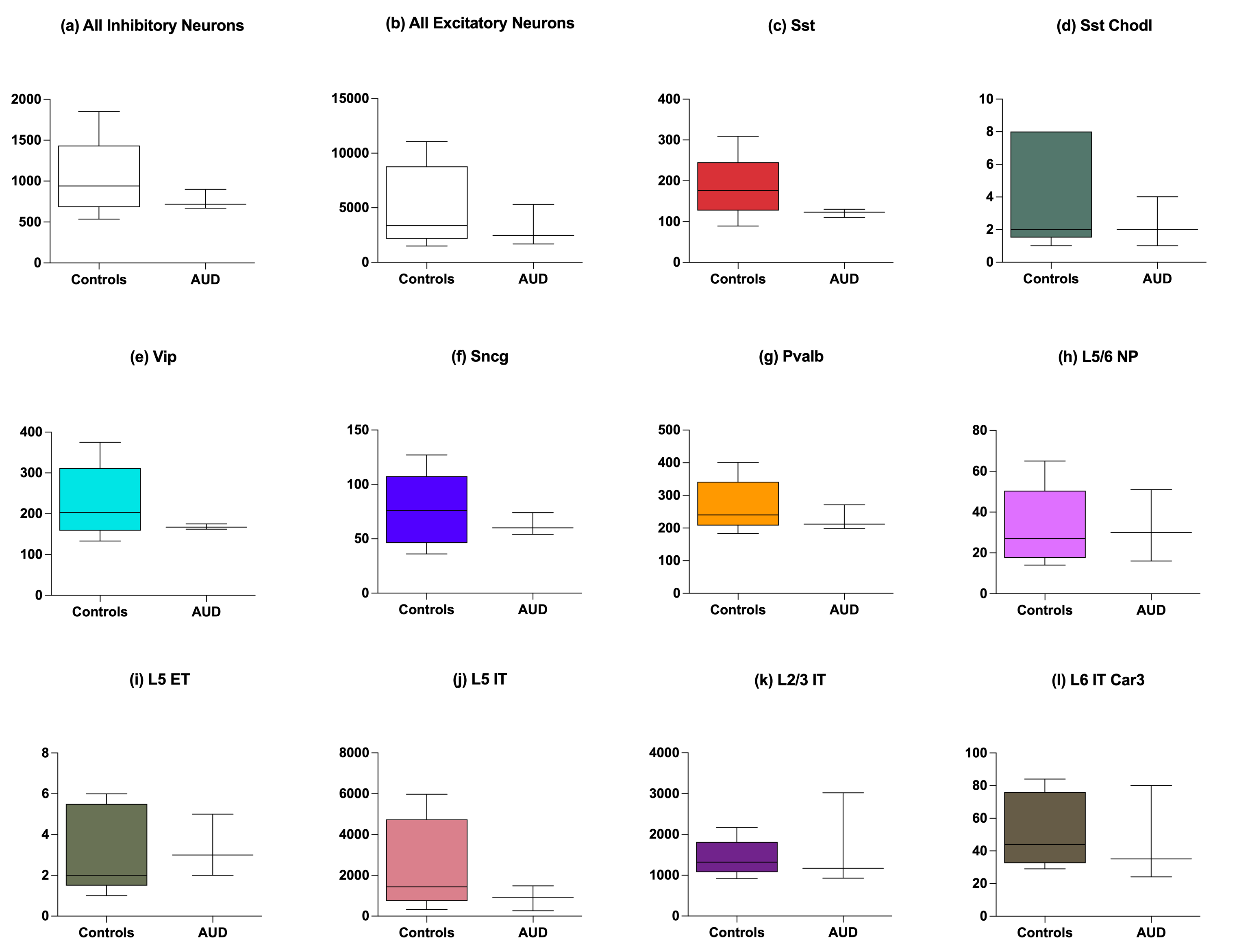

Supplement: Figure 2-2 — Distribution of number of neurons in the corresponding cell-types in each donor category in AUD data: (a) All Inhibitory Neurons, there is no significant difference between groups (t-test, t(6) = 0.9154, p = 0.3953). (b) All Excitatory Neurons, there is no significant difference between groups (t-test, t(6) = 0.7860, p = 0.4618). (c) Sst, there is no significant difference between groups (t-test, t(6) = 1.333, p = 0.2309). (d) Sst Chodl, there is no significant difference between groups (t-test, t(6) = 0.8563, p = 0.4247). (e) Vip, there is no significant difference between groups (t-test, t(6) = 1.111, p = 0.3093). (f) Sncg, there is no significant difference between groups (t-test, t(6) = 0.6640, p = 0.5314). (g) Pvalb, there is no significant difference between groups (t-test, t(6) = 0.7872, p = 0.4611). (h) L5/6 NP, there is no significant difference between groups (t-test, t(6) = 0.01910, p = 0.9854). (i) L5 ET, there is no significant difference between groups (t-test, t(6) = 0.09232, p = 0.9295). (j) L5 IT, there is no significant difference between groups (t-test, t(6) = 1.152, p = 0.2931). (k) L2/3 IT, there is no significant difference between groups (t-test, t(6) = 0.5118, p = 0.6271). (l) L6 IT Car3, there is no significant difference between groups (t-test, t(6) = 0.3155, p = 0.7631). Download Figure 2-2, TIF file. [file eneuro-11-ENEURO.0118-24.2024-s002.tif]

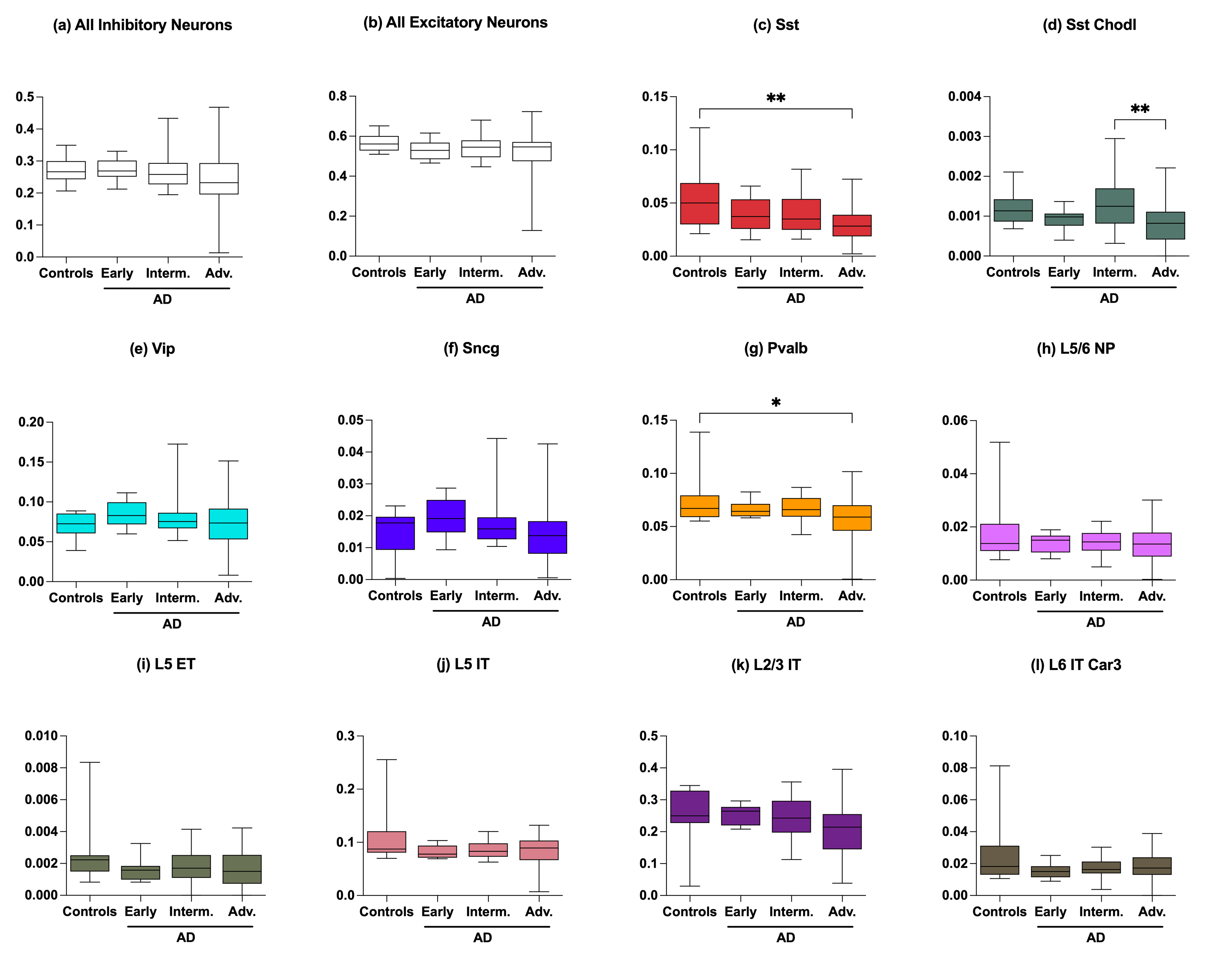

Supplement: Figure 3-1 — Distribution of neuronal proportions in the corresponding neuron types in each donor category in SEA-AD data: (a) All Inhibitory Neurons, there is no significant difference between groups (One-way ANOVA, F(3,81) = 1.053, p = 0.3738). (b) All Excitatory Neurons, there is no significant difference between groups (One-way ANOVA, F(3,81) = 1.339, p = 0.2675). (c) Sst, One-way ANOVA revealed a significant difference between groups (F(3,81) = 4.381, p = 0.0066). **p < 0.01, vs Controls; Tukey's test. (d) Sst Chodl, One-way ANOVA revealed a significant difference between groups (F(3,81) = 4.956, p = 0.0033). **p < 0.01, vs Controls; Tukey's test. (e) Vip, there is no significant difference between groups (One-way ANOVA, F(3,81) = 0.9486, p = 0.4212). (f) Sncg, there is no significant difference between groups (One-way ANOVA, F(3,81) = 1.523, p = 0.2148). (g) Pvalb, One-way ANOVA revealed a significant difference between groups (F(3,81) = 3.697, p = 0.0151). *p < 0.05, vs Controls; Tukey's test. (h) L5/6 NP, there is no significant difference between groups (One-way ANOVA, F(3,81)= 1.168, p = 0.3271). (i) L5 ET, there is no significant difference between groups (One-way ANOVA, F(3,81) = 1.936, p = 0.1303). (j) L5 IT, there is no significant difference between groups (One-way ANOVA, F(3,81) = 2.266, p = 0.0870). (k) L2/3 IT, there is no significant difference between groups (One-way ANOVA, F(3,81) = 2.543, p = 0.0620). (l) L6 IT Car3, there is no significant difference between groups (One-way ANOVA, F(3,81) = 2.377, p = 0.0760). Download Figure 3-1, TIF file. [file eneuro-11-ENEURO.0118-24.2024-s003.tif]

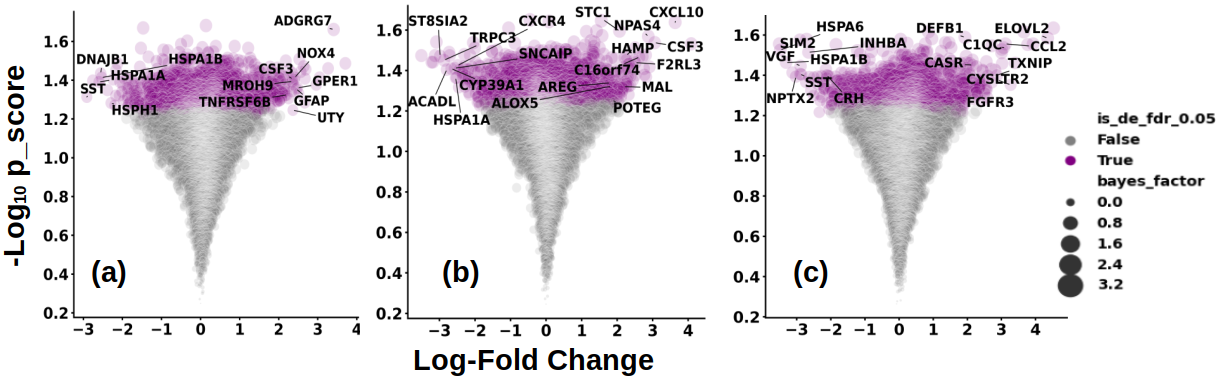

Supplement: Figure 4-1 — Volcano plots of some selected DEGs in the three AD stages: (a) Early AD, (b) Intermediate AD, (c) Advanced AD. Download Figure 4-1, TIF file. [file eneuro-11-ENEURO.0118-24.2024-s004.tif]

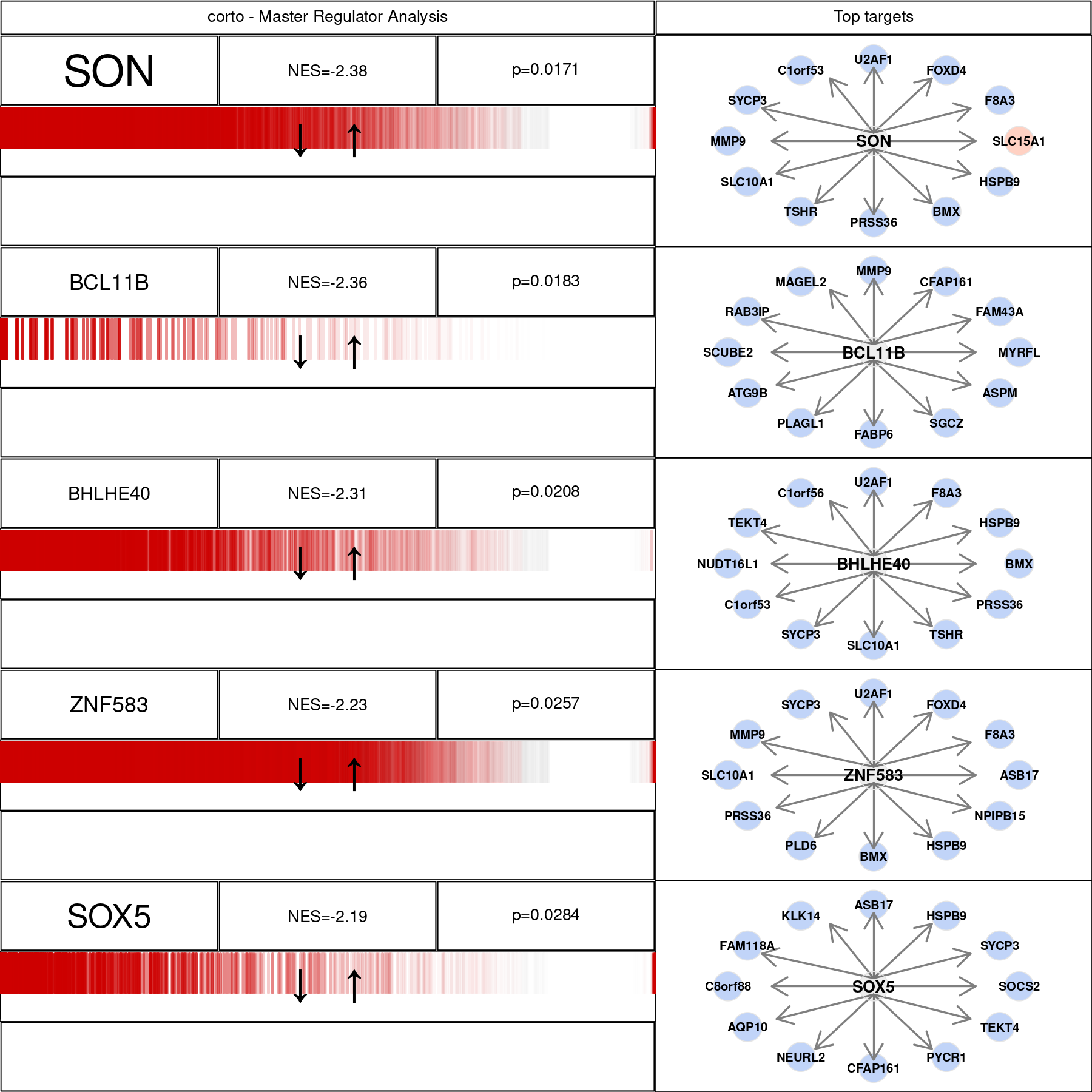

Supplement: Figure 11-1 — Master Regulator Analysis of the significantly differentially expressed transcription factors in transcription profiles of AUD samples in early AD. Genes on the left, are the selected transcription factors found dysregulated in AUD, their transcriptional signature from early AD is compared with controls. Download Figure 11-1, TIF file. [file eneuro-11-ENEURO.0118-24.2024-s008.tif]

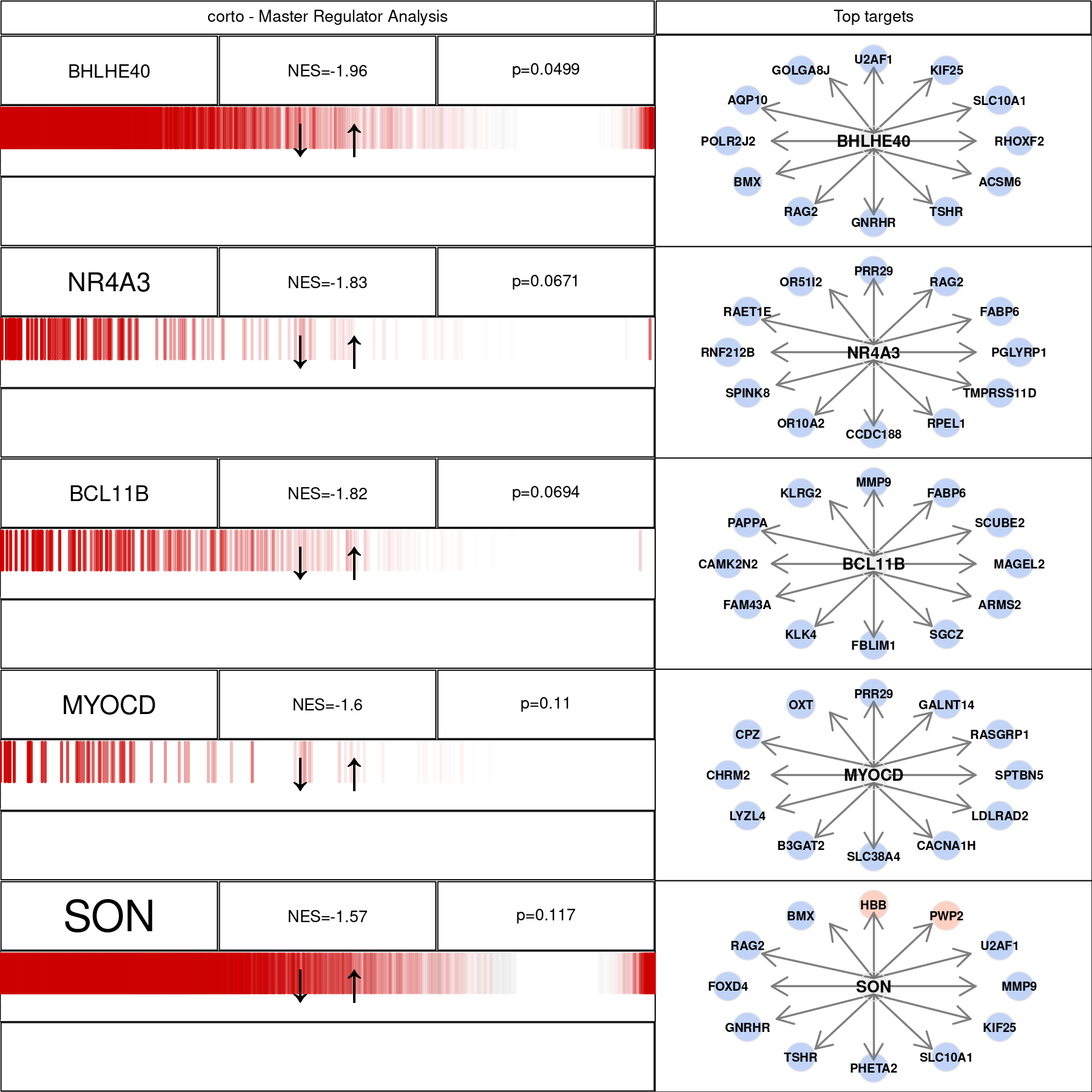

Supplement: Figure 11-2 — Master Regulator Analysis of the significantly differentially expressed transcription factors in transcription profiles of AUD samples in intermediate AD. Genes on the left, are the selected transcription factors found dysregulated in AUD, their transcriptional signature from intermediate AD is compared with controls. Download Figure 11-2, TIF file. [file eneuro-11-ENEURO.0118-24.2024-s009.tif]
